# Orthogonal Ionic Liquid‐Based Extraction Strategy Enables Amyloid‐Specific Profiling of Aggregate Proteome

**DOI:** 10.1002/advs.202523685

**Published:** 2026-04-20

**Authors:** Shiying Zheng, Ye Liu, Bowen Zhong, Huiying Chu, Zhou Gong, Baofeng Zhao, Mengchun Cheng, Zhen Liang, Yukui Zhang, Qun Zhao, Lihua Zhang

**Affiliations:** ^1^ State Key Laboratory of Medical Proteomics National Chromatographic R. & A. Center Dalian Institute of Chemical Physics Chinese Academy of Sciences Dalian China; ^2^ University of Chinese Academy of Sciences Beijing China; ^3^ Interdisciplinary Research Center for Biology and Chemistry Liaoning Normal University Dalian China; ^4^ School of Chemistry and Material Science University of Science and Technology of China Hefei China; ^5^ State Key Laboratory of Magnetic Resonance and Atomic Molecular Physics Innovation Academy for Precision Measurement Science and Technolog, Chinese Academy of Sciences Wuhan China

**Keywords:** amyloid deposits, Ionic liquids, neurodegenerative diseases, orthogonal extraction, proteome

## Abstract

Amyloid deposits, composed of insoluble cross‐β‐sheet fibrils, are pathological hallmarks of many neurodegenerative diseases. However, their structural heterogeneity and extreme insolubility hinder characterization of their molecular composition and deposition mechanisms. Here, an orthogonal ionic liquid‐based extraction strategy (Orth‐*i*EA) was developed to selectively enrich amyloid aggregates from complex biological samples. Systematic screening identified tetramethylguanidine tetrafluoroborate (TMGBF_4_) as a potent ionic liquid capable of disrupting hydrogen bonds within cross‐β‐sheet structures, thereby solubilizing amyloid aggregates. In contrast, 1‐dodecyl‐3‐methylimidazolium chloride (C12ImCl) preferentially solubilized non‐amyloid proteins through hydrophobic interactions. In combination, these two reagents constitute the orthogonal extraction system, enabling the highly selective enrichment of amyloid aggregates from complex biological samples while remaining compatible with downstream LC‐MS/MS proteomic workflows after mild desalting. Application of Orth‐*i*EA to hippocampal tissue from 12‐month‐old 3xTg Alzheimer's disease model mice identified numerous amyloid‐associated proteins enriched in mitochondrial components, which was further confirmed by immunofluorescence co‐localization analysis. Functional and network analyses converged on pathways involving protein transport, mitochondrial translation, intracellular signaling, and apoptosis, revealing previously unrecognized molecular links between amyloid pathology and Alzheimer's disease. Therefore, Orth‐*i*EA provides a versatile chemical platform for selective isolation and molecular profiling of amyloid aggregates.

## Graphical abstract

An orthogonal ionic‐liquid extraction (Orth‐*i*EA) enables selective isolation of amyloid fibrils. TMGBF_4_ disrupts hydrogen‐bonded β‐sheet networks to solubilize amyloid aggregates, whereas C12ImCl interacts with hydrophobic regions of non‐amyloid proteins. The orthogonal extraction mechanisms provide improved selectivity and deeper proteome coverage from amyloid‐focused proteomics.

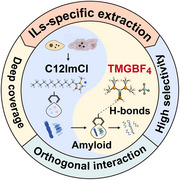

## 1 Introduction

Amyloid deposits are insoluble aggregates of misfolded proteins that typically exhibit fibrillar structures characterized by cross‐β‐sheet stacking [1, 2]. These aggregates are key histopathological markers of many neurodegenerative disorders, including Aβ plaques and Tau tangles in Alzheimer's disease (AD) [3, 4], α‐synuclein Lewy bodies in Parkinson's disease (PD) [5], and aberrant TDP‐43 aggregates in amyotrophic lateral sclerosis (ALS) [6]. By disrupting cellular homeostasis and triggering pathological cascades such as neuroinflammation and oxidative stress, amyloid deposits contribute to disease progression [4, 7]. Importantly, the molecular composition of amyloid deposits is heterogeneous and evolves during disease progression, directly impacting pathogenic mechanisms [8]. Therefore, precise characterization of amyloid composition is critical for elucidating disease pathogenesis, identifying specific biomarkers, and developing conformation‐targeted diagnostic and therapeutic strategies.

A variety of techniques have been employed to detect and study amyloid aggregates. Imaging modalities such as positron emission tomography can provide spatial distribution in vivo [9], while histochemical stains, including Congo red or Thioflavin‐T (ThT), facilitate visualization at the tissue level [10, 11]. Structural biology approaches such as nuclear magnetic resonance (NMR) and cryo‐electron microscopy (cryo‐EM) provide detailed insights into fibril architecture [12, 13]. However, these methods cannot provide quantitative and comprehensive identification of amyloid composition at the proteome‐wide scale. This limitation restricts a full understanding of the molecular mechanisms underlying disease onset and progression, as well as the development of effective therapeutic interventions.

Proteomic approaches have therefore been used to characterize aggregated proteomes. Centrifugal enrichment coupled with liquid chromatography‐mass spectrometry (LC‐MS) is widely employed. For instance, sucrose density‐gradient ultracentrifugation combined with ultrasonication has enabled the identification of novel Aβ42‐interacting proteins involved in amyloid formation [14]. Differential centrifugation with tandem mass tag (TMT)‐based MS has revealed detergent‐insoluble proteins enriched in postmortem AD brain tissues [15]. However, these methods often co‐isolate non‐amyloid proteins with similar densities, reducing the specificity of amyloid protein identification.

Targeted approaches have been developed to capture amyloid aggregates. Immunohistochemistry combined with laser microdissection‐MS enables spatial localization and analysis of aggregates within tissue [16, 17]. However, these methods inevitably collect proteins located near the inclusions, including proteins that are not structurally incorporated into amyloid fibrils, thereby limiting extraction specificity. Proximity labeling‐MS techniques, such as BioID and APEX2, have been used to label and enrich proteins associated with amyloid aggregates [18, 19]. For instance, BioID has been used to map the interactome of aggregated TDP‐43, providing insights into nuclear‐cytoplasmic transport defects in ALS and frontotemporal dementia (FTD) [18]. Nevertheless, these methods cannot reliably distinguish interactions with aggregated proteins from their native counterparts, potentially confounding aggregate‐specific interaction maps. Similarly, small‐molecule probe‐based proximity labeling approaches can capture broader aggregated proteomes but lack sufficient selectivity for amyloid‐specific interactions [20].

Because amyloid fibrils are highly insoluble and structurally rigid, their efficient extraction requires reagents capable of disrupting the extensive hydrogen‐bond network that stabilizes cross‐β‐sheet structures. Conventional denaturants and detergents, such as urea and sodium dodecyl sulfate (SDS), operate through non‐specific mechanisms and solubilize a wide range of insoluble protein complexes. These reagents often fail to release low‐abundance or tightly embedded amyloid components efficiently. Therefore, efficient and selective extraction requires reagents capable of penetrating the dense cross‐β‐sheet hydrogen bond network while discriminating amyloids from other insoluble assemblies.

Ionic liquids (ILs), which are tunable organic salts [21], offer adjustable polarity, hydrogen bonding capability, and hydrotropic or hydrophobic properties, allowing the design of reagents with distinct extraction mechanisms such as those targeting membrane proteins [22, 23] and nucleic acids [24]. This versatility suggests that ILs may provide a promising yet underexplored strategy for selective amyloid solubilization.

In this study, ILs with diverse cation‐anion combinations were systematically screened to identify reagents capable of selectively solubilizing amyloid aggregates. Tetramethylguanidine tetrafluoroborate (TMGBF_4_, C_5_H_14_BF_4_N_3_) was identified as a highly effective chaotrope for solubilizing amyloid aggregates via disruption of hydrogen‐bond networks within cross‐β‐sheet structures. In contrast, the previously well‐characterized IL 1‐dodecyl‐3‐methylimidazolium chloride (C12ImCl, C_16_H_31_ClN_2_) selectively solubilized non‐amyloid proteins through hydrophobic interactions. Sequential application of these reagents enabled the development of an orthogonal IL‐based extraction (Orth‐*i*EA) approach with high amyloid specificity while remaining compatible with LC‐MS‐based proteomics workflows. Application of Orth‐*i*EA to hippocampal tissue from 12‐month‐old 3xTg AD mice enabled targeted identification of amyloid‐associated proteins, which was further supported by immunofluorescence co‐localization analysis. Overall, the Orth‐*i*EA approach constitutes an efficient and versatile platform for the selective isolation and comprehensive molecular profiling of amyloid aggregates from complex biological matrices, thereby providing valuable insights into the mechanisms and biomarkers of neurodegenerative diseases.

## 2 Results and Discussion

### 2.1 Systematic Screening and Identification of ILs for Specific Extraction of Amyloid Aggregates

**FIGURE 1:**
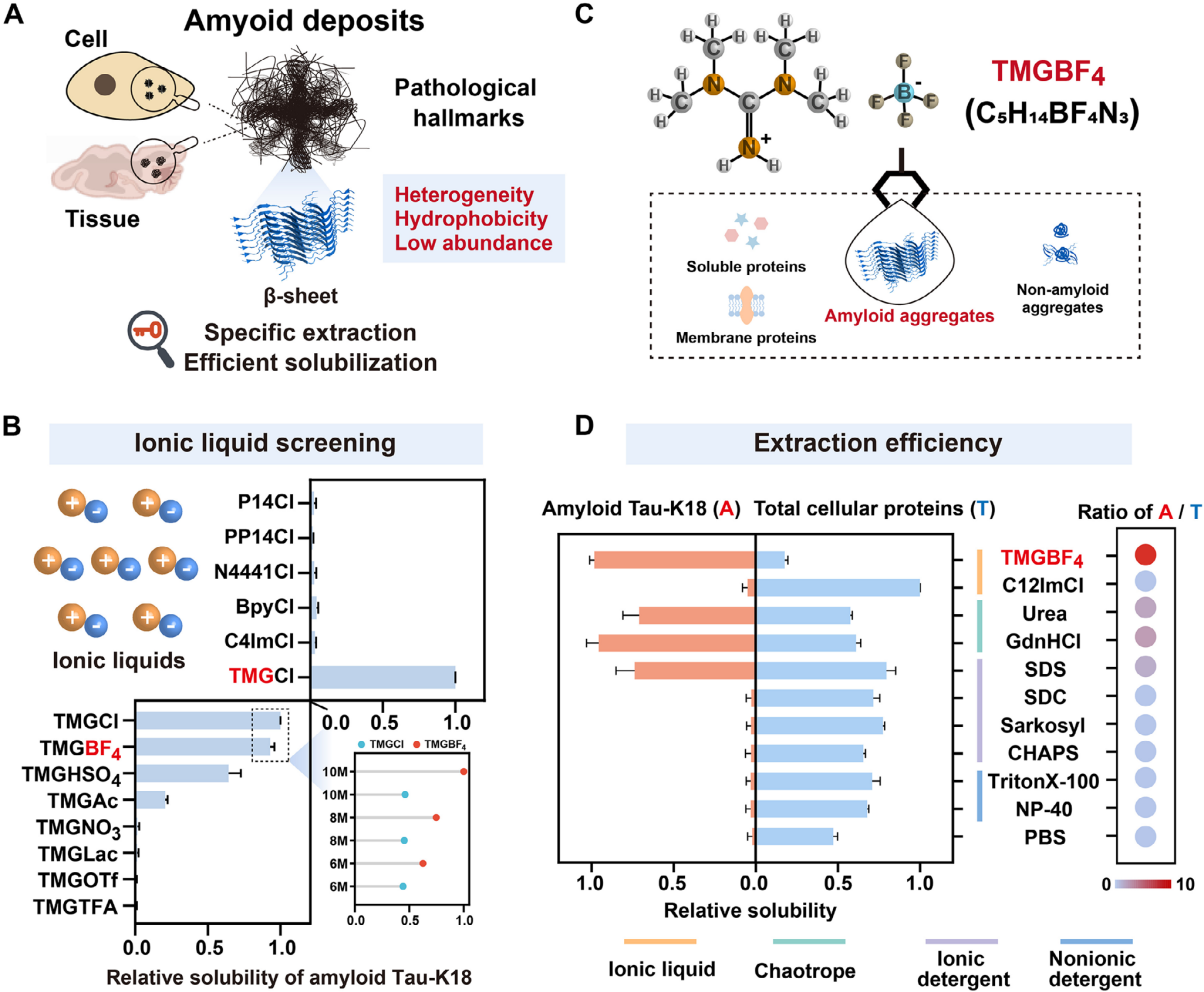
Systematic Screening of ILs for Specific Extraction of Amyloid Aggregates. (A) Key challenges in extracting amyloid aggregates from complex biological samples. (B) Systematic screening of ILs using amyloid Tau‐K18: evaluation of cations, anions, and IL concentrations. In screening of cations and anions, concentration of ILs was fixed at 1mol·L^−1^. Details of the ILs used are provided in Table S1. Bottom right panel shows the concentration‐dependent solubilization of amyloid Tau‐K18 by TMGCl and TMGBF_4_. (C) Ball‐and‐stick molecular structure and chemical formula of TMGBF_4_ and its specific solubilization to amyloid aggregates. (D) Solubility comparison of ILs and conventional solvents for total cellular proteins and amyloid Tau‐K18. Relative solubility of amyloid Tau‐K18 (A, orange bars) and total cellular proteins (T, blue bars) was measured for each solvent. The ratio of amyloid Tau‐K18 to total cellular protein extracted by each solvent (A/T) is shown on the right. Solvents are categorized as ILs (C12ImCl, 10% w/v; TMGBF_4_, 10 mol·L^−1^), Chaotropes (urea, 8 mol·L^−1^; GdnHCl, 6 mol·L^−1^), Ionic detergents (SDS, 4% w/v; SDC, Sarkosyl, and CHAPS, 1%w/v each) and Nonionic detergents (Triton X‐100, NP‐40, 1% v/v each). Protein solubility was normalized to the maximum value observed among all tested solvents and is presented as relative solubility (mean ± SD; n = 3–4).

Amyloid deposits are characterized by their conformational heterogeneity, extensive cross‐β‐sheet hydrogen bond networks, extreme insolubility, and often low abundance. These features present significant challenges for their selective extraction from complex proteomes (Figure 1A). ILs, which possess tunable physicochemical properties arising from diverse cation‐anion combinations, provide a promising alternative to mediate electrostatic interactions, hydrogen bonding, hydrophobic effects, and van der Waals forces [25], indicating that ILs could offer a more selective means of destabilizing amyloid fibrils while sparing non‐amyloid proteins.

To evaluate this possibility, diverse ILs were systematically screened based on different cation‐anion combinations. Tau‐K18, a truncated Tau construct containing the amyloidogenic core region [26], was used as a model amyloid system to quantify solubilization efficiency under standardized conditions. Among six cation scaffolds tested (Table S1), ILs containing the tetramethylguanidine (TMG) cation showed superior solubility for amyloid Tau‐K18. Considering the significant role of anions, we screened eight water‐soluble TMG‐based ILs, identifying TMG tetrafluoroborate (TMGBF_4_) and TMG chloride (TMGCl) as exceptional solubilizers of amyloid Tau‐K18 (Figure 1B and Table S1). Concentration‐dependent experiments further demonstrated that 10 mol·L^−1^ TMGBF_4_ exhibited the highest solubility, exceeding that of TMGCl at the same concentration (Figure 1B).

To evaluate the extraction selectivity of TMGBF_4_, we compared its ability to solubilize amyloid Tau‐K18 and total cellular proteins (Figure 1C, Figure S1). TMGBF_4_ efficiently solubilized amyloid Tau‐K18 while exhibiting limited extraction of total cellular proteins, resulting in the highest amyloid‐to‐total protein extraction ratio among all tested solvents, which demonstrated its superiority in amyloid enrichment. In contrast, C12ImCl solubilized a wider spectrum of proteins but was less efficient for amyloid extraction, indicating that the two reagents may play orthogonal roles in protein extraction. Conventional extraction reagents, including urea, guanidine hydrochloride (GdnHCl), and SDS, indiscriminately solubilized both amyloids and non‐amyloid proteins, whereas mild ionic detergents (SDC, Sarkosyl, CHAPS) and nonionic detergents (Triton X‐100, NP‐40) displayed broader but less efficient solubilization profiles (Figure 1D). Collectively, these results suggest that combining reagents with orthogonal selectivity may improve both the yield and specificity of amyloid isolation.

To further validate the selectivity of TMGBF_4_, we examined its effect on α‐synuclein aggregates (Figure S2). TMGBF_4_ efficiently disrupted α‑synuclein fibrils, as evidenced by pronounced quenching of ThT fluorescence, a marker of β‑sheet content. In contrast, C12ImCl preferentially extracted non‑amyloid aggregates while largely preserving ThT fluorescence. These results further support TMGBF_4_ as a potent and selective solubilizer for amyloid fibrils.

### 2.2 Mechanistic Insights for Selective Amyloid Solubilization

**FIGURE 2:**
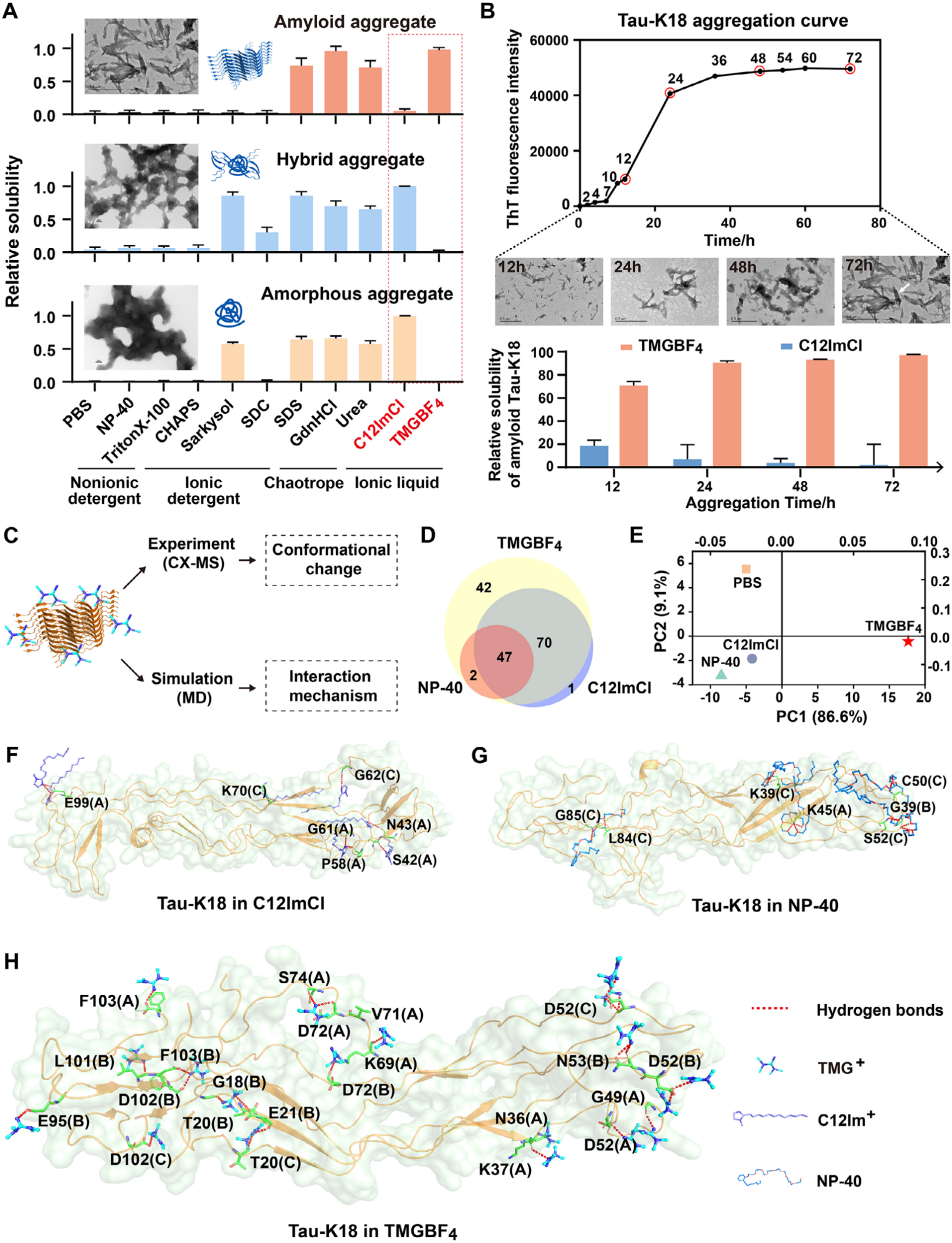
Comparative solubilization profiles and mechanistic insights of ILs and other solvents toward protein aggregates of distinct morphologies. (A) Solubility comparison of ILs and conventional solvents for protein aggregates of distinct morphologies. Representative TEM images of Tau‐K18 (amyloid), TTR (hybrid), and DHFR (amorphous) aggregates are shown. Scale bars: 0.5 µm for Tau‐K18; 100 nm for TTR and DHFR. Solvents are categorized as ILs (C12ImCl, 10% w/v; TMGBF_4_, 10 mol·L^−1^), Chaotropes (urea, 8 mol·L^−1^; GdnHCl, 6 mol·L^−1^), Ionic detergents (SDS, 4% w/v; SDC, Sarkosyl, and CHAPS, 1%w/v each) and Nonionic detergents (Triton X‐100, NP‐40, 1% v/v each). Protein solubility was normalized to the maximum value obtained among the tested solvents and is presented as relative solubility (mean ± SD; n = 3). (B) Solubilization of Tau‐K18 aggregates by C12ImCl and TMGBF_4_ at different aggregation stages. Tau‐K18 aggregation was monitored by ThT fluorescence assay and TEM images were captured at different aggregation stages of 12, 24, 48, and 72 h. Scale bar: 0.5 µm. Relative solubility was calculated as in (A) and is expressed as mean ± SD (n = 3). (C) Combined CX‐MS and MD simulations investigating solvent‐specific solubilization mechanisms. (D) Venn diagram showing cross‐linked residue pairs in Tau‐K18 solubilized by NP‐40, C12ImCl, and TMGBF_4_. (E) Principal component analysis of cross‐linked sites and their spectral counts for Tau‐K18 in PBS, NP‐40, C12ImCl, and TMGBF_4_. (F‐H) MD simulations showing solvent interactions with the Tau‐K18 trimer. Tau‐K18 structure (PDB ID: 8Q9A) is a homotrimer with three individual chains (labeled A, B, and C). Snapshots show Tau‐K18 trimer interacting with C12ImCl (F), NP‐40 (G), and TMGBF_4_ (H). Tau‐K18 protein is shown as an orange cartoon with a pale green surface, and interacting residues are shown as green sticks. Solvent molecules are colored as follows: NP‐40 (marine), C12ImCl (light blue), TMGBF_4_ (cyan). Hydrogen bonds are indicated by red dashed lines.

Given the structural heterogeneity of protein aggregates, we extended our solubilization assessment to three aggregate types: amorphous aggregates (e.g., dihydrofolate reductase, DHFR), hybrid aggregates with amorphous cores and peripheral fibrils (e.g., transthyretin, TTR), and canonical amyloid fibrils (e.g., Tau‐K18) (Figure 2A). Distinct selectivity patterns were observed across solvent classes. Among the ILs, TMGBF_4_ showed a strong preference for amyloid fibrils, whereas C12ImCl displayed broader activity toward both amorphous and hybrid aggregates. Conventional extraction reagents (urea, GdnHCl, SDS) solubilized all aggregate types non‐selectively, while mild detergents (e.g., NP‐40, Triton X‐100) were generally ineffective. Overall, these results underscore that IL composition critically shapes extraction selectivity, with TMGBF_4_ standing out as the most amyloid‑biased solubilizer in this set.

Building on the observation that TMGBF_4_ preferentially solubilizes mature amyloid fibrils, we further investigated how aggregate properties evolve during amyloidogenesis and how this influences extraction selectivity. Tau‐K18 samples were collected at four aggregation time points (12, 24, 48, and 72 h), and aggregated species were enriched using TMGBF_4_ (Figure 2B). TEM confirmed the structural progression from disordered lamellar intermediates to well‐formed fibrils. Solubility assays showed that the proportion of aggregates extractable by C12ImCl declined steadily over time, consistent with the increasing amyloid content, whereas TMGBF_4_ extracted progressively greater amounts of aggregated protein. These results support the idea that TMGBF_4_ exhibits a pronounced preference for later‐stage amyloid aggregates, motivating further experiments to probe the molecular determinants of this selectivity.

To elucidate the molecular basis of this selectivity, we combined chemical cross‐linking mass spectrometry (CX‐MS) with molecular dynamics (MD) simulations (Figure 2C). CX‐MS experiments were performed to analyze conformational changes in Tau‐K18 in its native state (PBS) and in amyloid states solubilized with NP‐40, C12ImCl, or TMGBF_4_ (Figure S3A). Three amine‐reactive cross‐linkers with distinct spacer lengths and backbone chemistries were used to characterize lysine residue accessibility (Figure S3B). Cross‐linking results showed that TMGBF_4_‐solubilized Tau‐K18 exhibited the largest number of labeled lysine residues, indicating enhanced solvent exposure and conformational unfolding (Figure S3C,D). The Venn diagram (Figure 2D) further demonstrated that TMGBF_4_ included nearly all cross‐linked sites identified in NP‐40 and C12ImCl, while additionally revealing 42 unique sites, suggesting the presence of multiple unfolded conformers in TMGBF_4_. Principal component analysis further confirmed a distinct conformational profile of amyloid Tau‐K18 in TMGBF_4_ compared with the other solvents (Figure 2E).

MD simulations provided complementary structural insight into solvent‐protein interactions. All solvents tested—NP‐40, C12ImCl, and TMGBF_4_—destabilized the Tau‐K18 trimer by reducing binding free energy between its A and B/C chains (Table S2). Among them, TMGBF_4_ exhibited the most pronounced destabilizing effect, driven by extensive hydrogen‐bonding interactions with the Tau‐K18 trimer (Figure 2F–H). Specifically, C12ImCl (Figure 2F) and NP‐40 (Figure 2G) primarily interacted with terminal residues of the trimer, forming fewer hydrogen bonds and exhibiting limited impact on β‐sheet integrity. In contrast, TMGBF_4_ (Figure 2H) disrupted key inter‐chain hydrogen bonds within critical β‐sheet layers involving residues D52, N53, L101, D102, and F103. Additionally, TMGBF_4_ stably interacted with central residues, including K69, V71, D72, and S74, thereby further destabilizing the trimeric core.

Our previous work demonstrated that the long alkyl chain of C12ImCl enhances its solubilization capacity for transmembrane proteins, outperforming conventional detergents such as SDS [27, 28]. MD simulations further revealed that the long alkyl chain, rather than the imidazolium head group, preferentially adsorbs onto hydrophobic amino acid surfaces, thereby facilitating membrane‑protein solubilization through dominant hydrophobic interactions [22]. This hydrophobic‑driven mechanism differs fundamentally from that of TMGBF_4_, which solubilizes amyloid fibrils primarily through disruption of internal hydrogen‑bonded β‑sheet networks. Together, these findings highlight the distinct yet complementary interaction mechanisms of the two ILs.

Collectively, our results demonstrate that TMGBF_4_ and C12ImCl operate through complementary mechanisms: TMGBF_4_ solubilizes amyloid fibrils by disrupting internal hydrogen‐bonded β‐sheet networks, whereas C12ImCl targets hydrophobic surface regions of non‐amyloid or amorphous aggregates. This synergy enables efficient and selective separation of structurally diverse aggregates from complex biological matrices, thereby supporting accurate subtyping of amyloid species and facilitating in‐depth investigation of amyloid‐associated pathology in neurodegenerative diseases.

### 2.3 Development of an Orthogonal Extraction System for Selective Amyloid Enrichment in Complex Biological Samples

**FIGURE 3:**
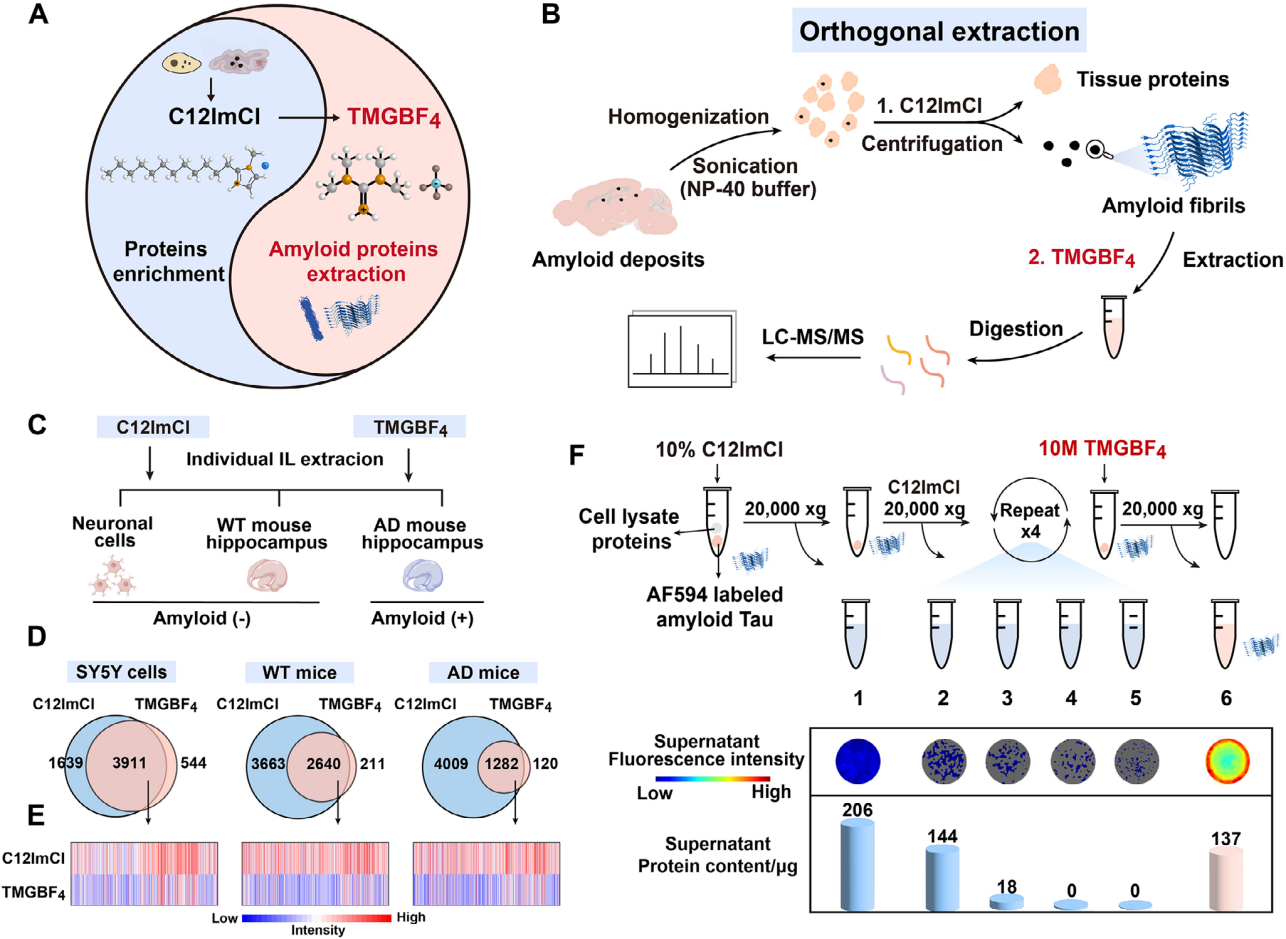
Development of the Orth‐*i*EA Strategy. (A) Conceptual overview of the Orth‐*i*EA workflow. In this strategy, C12ImCl is first used to selectively solubilize and remove soluble proteins and non‐amyloid aggregates, thereby enriching the sample for insoluble amyloid components. TMGBF_4_ is subsequently applied to efficiently solubilize and recover amyloid fibrils. (B) Schematic illustration of amyloid deposits profiling in tissue using the Orth‐*i*EA strategy. (C) Individual extractions using C12ImCl or TMGBF_4_ were performed in amyloid‑negative systems (SH‑SY5Y cells and wild‐type mouse hippocampus) and an amyloid‑positive system (3xTg‑AD mouse hippocampus). (D) Venn diagrams showing proteins identified by C12ImCl and TMGBF_4_ extractions across the three systems. (E) Heatmaps showing intensity values for proteins identified in both IL extracts. (F) Performance Evaluation of Orth‐*i*EA Strategy using a spike‐in model. Fluorescence‐labeled Tau‐K18 amyloid fibrils were added to whole‐cell lysates. Following extraction with 10% (w/v) C12ImCl and repeated washing, soluble components were removed. The remaining insoluble fraction was subsequently solubilized using 10 mol·L^−1^ (10 ^m^ ) TMGBF_4_. Supernatant analysis showed minimal protein and fluorescence signals during C12ImCl treatment, whereas both signals increased markedly after TMGBF_4_ extraction, indicating efficient recovery of amyloid fibrils.

Based on the insights into their distinct mechanisms, we integrated the optimized ILs into an orthogonal two‑step extraction strategy configured for high‑fidelity amyloid proteomics (Orth‐*i*EA, Figure 3A). In the first step, C12ImCl selectively solubilizes and removes soluble proteins and non‑amyloid aggregates, thereby reducing background complexity and enriching the remaining fraction for insoluble components. In the second step, TMGBF_4_ efficiently disrupts the β‑sheet architecture and solubilizes the residual amyloid fibrils that are typically resistant to conventional extraction reagents. This sequential extraction design achieves both depletion of non‑amyloid materials and highly selective recovery of low‑abundance amyloid aggregates (Figure 3B).

To validate the complementary solubilization behaviors of the ILs, C12ImCl and TMGBF_4_ extractions were independently applied to three biological systems: two amyloid‑negative systems (SH‑SY5Y cells and wild‐type mouse hippocampus) and one amyloid‑positive system (hippocampus from 12‑month‑old 3xTg‑AD mice), followed by quantitative proteomic analysis (Figure 3C). Given that the amyloid‑associated fraction of most proteins represents only a small proportion of their total cellular abundance, detecting amyloid‑associated proteins in complex samples is challenging. Proteomic analysis revealed substantial overlap in proteins identified by both ILs across all systems, reflecting high MS detection sensitivity (Figure 3D). Quantitative comparisons showed that most shared proteins were more abundant in C12ImCl extracts, highlighting its efficiency in solubilizing non‑amyloid proteins (Figure 3E). In contrast, proteins enriched in TMGBF_4_ extracts from 3xTg‑AD mouse hippocampus exhibited higher predicted aggregation propensity than those from wild‑type tissue (average scores: AD = −1.23 vs. WT = −1.37, Dataset S1), consistent with the selective enrichment of amyloid‐associated proteins.

We further evaluated the Orth‐*i*EA strategy using a spike‐in model in which Alexa Fluor 594 (AF594)‐labeled Tau‐K18 amyloid fibrils were added to whole‐cell lysates (Figure 3F). The mixture was first extracted with 10% (w/v) C12ImCl and repeatedly washed to remove soluble components. Analysis of the supernatants showed a progressive decrease in total protein wash cycles, while the fluorescence signal of labeled Tau‐K18 remained largely unchanged, indicating minimal loss of amyloid material during the C12ImCl step. Subsequent treatment with TMGBF_4_ produced a marked increase in both fluorescence intensity and protein release, demonstrating efficient solubilization of the retained amyloid aggregates. Together, these results demonstrate that the Orth‐*i*EA method sequentially removes non‐amyloid proteins with C12ImCl and subsequently recovers amyloids with TMGBF_4_, thereby providing a robust and selective workflow for isolating amyloid assemblies from complex proteomic samples.

### 2.4 Proteomic Profiling of Amyloid Aggregates in 3xTg AD Mouse Hippocampus

**FIGURE 4:**
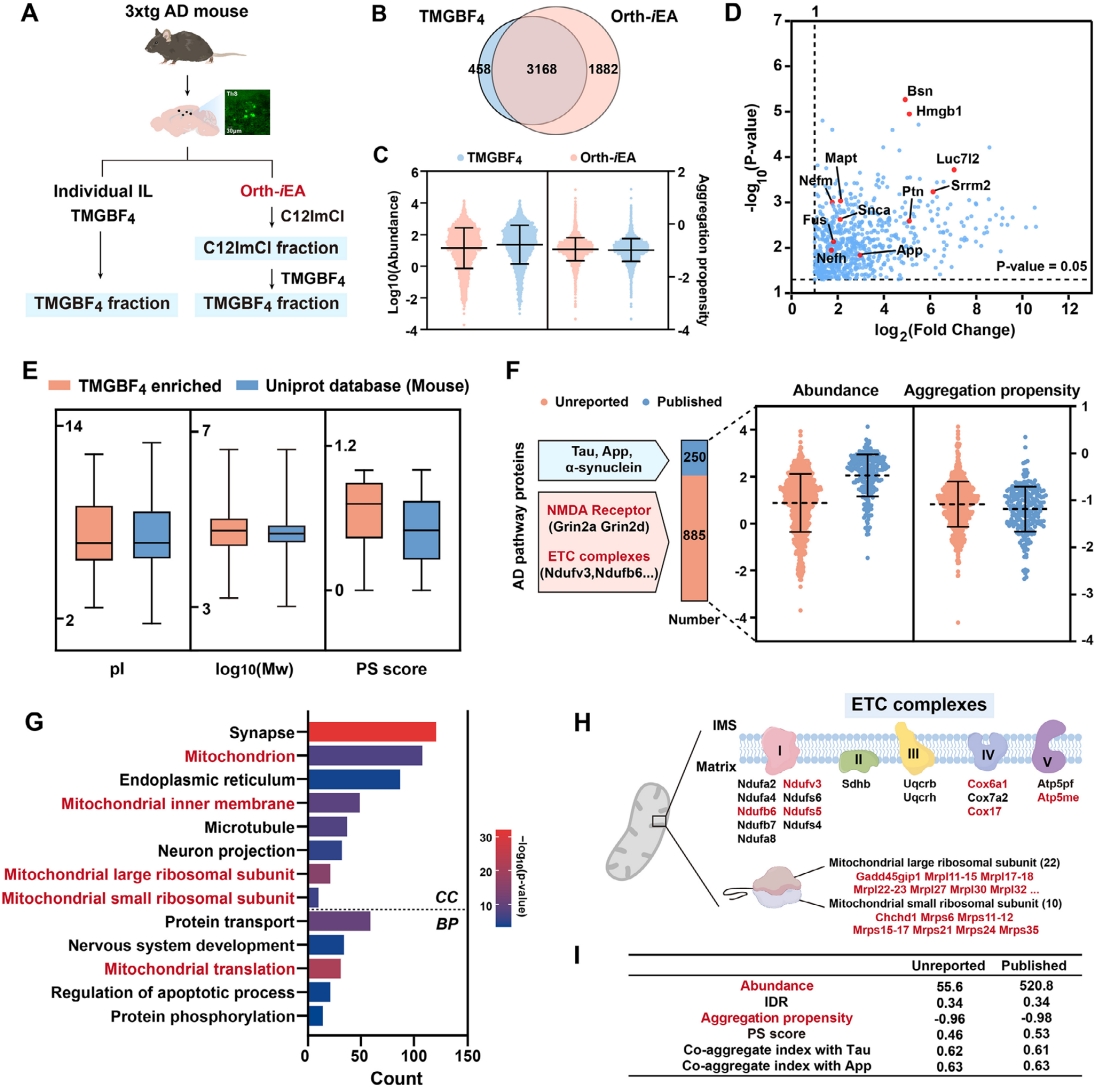
Application of Orth‐*i*EA to Amyloid Proteomic Profiling of the 3xTg‐AD Mouse Hippocampus. (A) Isolation of amyloid aggregates from the hippocampus of 12‐month‐old 3xTg‐AD mice using the individual TMGBF_4_ method and the Orth‐*i*EA strategy. The inset shows Thioflavin‐S (ThS)‐positive amyloid deposits in the hippocampus of the 12‐month‐old 3xTg‐AD mouse. Scale bar: 30 µm. (B) Venn diagram showing proteins identified using TMGBF_4_ extraction alone and the Orth‐*i*EA strategy. (C) Comparison of protein abundance and aggregation propensity for proteins identified by TMGBF_4_ extraction alone and the Orth‐*i*EA strategy. (D) Volcano plot showing proteins significantly enriched in the TMGBF_4_ fraction. Protein intensities from both TMGBF_4_ and C12ImCl fractions were compared across triplicates. Aggregation‐associated proteins are highlighted in red. *P*‐values were calculated by a two‐sided Student's *t*‐test, ^*^ *p* < 0.05. (E) Comparison of protein properties, including pI, Mw, and PS scores of the amyloid fraction proteins enriched by TMGBF_4_ with the mouse proteome database. (F) Comparison of abundance and aggregation propensity between newly identified amyloid fraction proteins and published proteins in the TMGBF_4_‐enriched dataset. The published data were derived from human postmortem AD brain tissue by differential centrifugal‐MS method [15], neurofibrillary tangles (NFTs) isolated by LCM‐MS methods [16, 17], and the NFT and amyloid plaque datasets from hippocampus in NeuroPro database [29]. (G) GO analysis of the newly identified amyloid fraction proteins in the TMGBF_4_‐enriched dataset, categorized by cellular component (CC) and biological process (BP). Mitochondrion‐related terms are highlighted in red. (H) Mapping of mitochondrial proteins identified in the TMBGF_4_‐enriched dataset onto ETC complexes and mitochondrial ribosomes. Newly identified proteins are indicated in red. (I) Comparison of abundance and aggregation‐related properties of the mitochondrial proteins in the TMGBF_4_‐enriched dataset, separated into newly identified and previously reported groups.

The Orth‐*i*EA strategy was further applied to hippocampal tissue from 12‐month‐old 3xTg AD mice to perform amyloid proteome analysis (Figure 4A). Clear differences were observed between proteins enriched by C12ImCl and those enriched by TMGBF_4_. A total of 1185 proteins were enriched (≥ 2 fold) in the TMGBF_4_ fraction, including well‐known amyloid‐associated proteins such as Tau, α‐synuclein, and App. By comparison, 1,888 proteins were enriched in the C12ImCl fraction, most of which were linked to membrane‐related processes (Figure S4). Physicochemical analysis confirmed that TMGBF_4_‐enriched proteins exhibited significantly higher phase separation (PS) scores, indicating stronger aggregation tendency, whereas C12ImCl‐enriched proteins displayed higher hydrophobicity (Grand Average of Hydropathy, GRAVY values) (Figure S4). These observations are consistent with the orthogonal extraction behaviors of the two ILs.

Moreover, the Orth‐*i*EA workflow identified more proteins and improved detection of low‑abundance species compared to TMGBF_4_ extraction alone (Figure 4B). Proteins isolated via Orth‐*i*EA also displayed higher predicted aggregation propensity (Figure 4C). This enhanced performance arises from the complementary action of C12ImCl, which removes abundant non‑amyloid components and reduces background complexity, thereby allowing TMGBF_4_ to profile the amyloid‑associated proteome with greater sensitivity and reliability.

After removing background proteins identified in the TMGBF_4_‐enriched proteome of 3‐month‐old wild‐type mouse hippocampus, a total of 1,135 proteins were found to be at least two‐fold more abundant in the TMGBF_4_ fraction than in the C12ImCl fraction, designated as amyloid fraction proteins (TMGBF_4_‐enriched dataset, Dataset S2). Among these, 727 proteins showed statistical significance (^*^ *p* < 0.05), including canonical amyloid‐related proteins such as Tau (Mapt), α‐synuclein (Snca), and App, as well as aggregation‐associated proteins such as Fus, Nefm, and Nefh (Figure 4D). Compared with the whole mouse proteome database, these enriched proteins exhibited similar distributions of isoelectric point (pI) and molecular weight (Mw), but noticeably higher average PS scores, indicative of greater intrinsic aggregation tendency under pathological conditions (Figure 4E).

To benchmark our results, we compared the TMGBF_4_‐enriched dataset with protein lists from previous amyloid proteomics studies, including differential centrifugation‐MS [15], LCM‐MS [16, 17], and the NeuroPro database [29]. Among them, 250 had been reported previously, whereas 885 had not appeared in earlier datasets. Notably, these newly identified proteins exhibited lower overall abundance but slightly higher predicted aggregation propensity, underscoring the superior sensitivity of the Orth‐*i*EA method in detecting low‐abundance, amyloid‐associated proteins that may have been overlooked in earlier studies. Pathway analysis revealed that 30 proteins from the enriched dataset were annotated in the KEGG Alzheimer's disease pathway, including 12 that have not previously been reported in amyloid proteomics studies. These proteins were mainly NMDA receptor subunits (Grin2a, Grin2b, Grin2d) and mitochondrial proteins involved in energy metabolism and the electron transport chain (ETC) (Figure 4F).

Gene Ontology (GO) analysis further showed significant enrichment of the newly identified proteins in cellular components such as mitochondria, synapses, and the endoplasmic reticulum, as well as biological processes including protein transport, mitochondrial translation, and regulation of the apoptotic process (Figure 4G). Within the TMGBF_4_‐enriched dataset, 146 proteins were annotated as mitochondrial, and 108 of these had not been previously associated with amyloid pathology. These proteins participate in critical mitochondrial functions such as translation, cellular respiration, and the regulation of oxidative stress‐induced cell death (Dataset S3). Among them, 17 proteins were components of the ETC, and 32 were mitochondrial ribosomal proteins (Figure 4H). Compared with previously reported proteins, these novel mitochondrial candidates exhibited comparable or greater aggregation propensities and PS scores, but were present at significantly lower abundance (Figure 4I).

Additionally, co‐aggregation analysis with Tau and App revealed strong aggregation correlations for both reported and newly identified mitochondrial proteins, indicating their potential roles in amyloid pathology (Figure 4I). Their dual involvement in mitochondrial translation and ETC function suggests they may mediate amyloid‐induced mitochondrial dysfunction—potentially impairing both energy production and protein synthesis, thereby exacerbating neurodegeneration.

In summary, the application of the Orth‐*i*EA strategy demonstrates its exceptional selectivity and sensitivity in isolating amyloid aggregates from complex tissues, uncovering a previously uncharacterized subset of aggregation‐prone proteins, particularly within the mitochondrial proteome. These results offer new molecular insights into the mechanisms of amyloid‐induced mitochondrial dysfunction and its role in neurodegenerative disease progression.

### 2.5 Validation and Network Analysis of Newly Identified Amyloid‐Associated Proteins

An expanded PPI network, integrating newly identified and previously reported proteins, showed that 73% of the newly identified proteins interacted with established amyloid‐associated factors (Dataset S4). This demonstrates their amyloid relevance, showcases Orth‐*i*EA's ability to connect known and novel pathological networks, and expands the amyloid interactome landscape.

**FIGURE 5:**
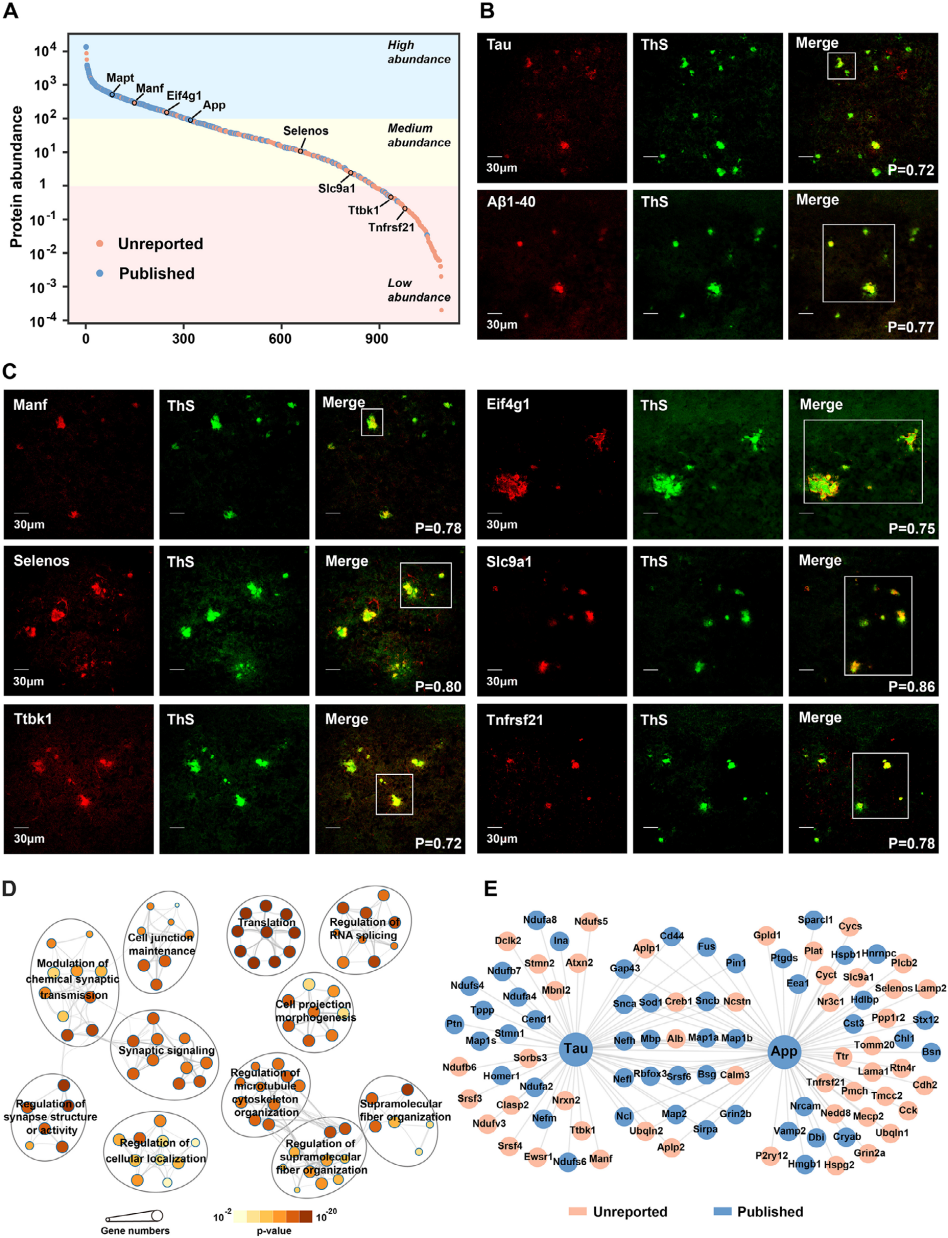
Validation and network analysis of amyloid‐associated proteins identified by the Orth‐*i*EA strategy. (A) Abundant distribution of amyloid‐associated proteins identified by Orth‐*i*EA. Previously reported and newly identified proteins are shown in blue and red, respectively. Protein abundance is displayed on a log_10_ scale. (B) Immunofluorescence co‐localization of canonical amyloid markers (Tau and Aβ1‐40) with ThS‐positive amyloid deposits in hippocampal tissue. Scale bar: 30 µm. (C) Immunofluorescence co‐localization validation of six newly identified amyloid‐associated proteins in the TMGBF_4_‐enriched dataset (Manf, Eif4g1, Selenos, Slc9a1, Ttbk1, and Tnfrsf21) with ThS‐positive amyloid deposits. Scale bar: 30 µm. (D) GO‐BP enrichment analysis of amyloid‐associated proteins identified in the TMGBF_4_‐enriched fraction. Circle size indicates the number of genes associated with each term, and color intensity reflects the corresponding p‐value. (E) PPI network centered on Tau and App for the amyloid‐associated proteins identified in the TMGBF_4_‐enriched fraction. Previously reported and newly identified proteins were represented in blue and red, respectively.

To validate the newly identified amyloid‐associated proteins identified by the Orth‐*i*EA strategy, we performed immunofluorescence staining on hippocampal tissue slices from 12‐month‐old 3xTg‐AD mice. Six previously unreported proteins, covering a dynamic abundance range spanning three orders of magnitude (Manf, Eif4g1, Selenos, Slc9a1, Ttbk1, and Tnfrsf21), were randomly selected from different abundance levels for further validation (Figure 5A). Thioflavin‐S (ThS) staining was used to specifically label amyloid deposits. Tau and Aβ1‐40, two canonical amyloid markers, served as positive controls and exhibited strong co‐localization with ThS, with Pearson correlation coefficients greater than 0.7 (Figure 5B). All 6 candidate proteins demonstrated significant co‐localization with amyloid deposits (Pearson coefficients ranging from 0.72 to 0.86), confirming their spatial association with amyloid pathology in situ (Figure 5C). Notably, these proteins span diverse functional categories. including synaptic maintenance (Manf) [30], regulation of Tau phosphorylation and aggregation (Ttbk1) [31], and neuroinflammatory cascades involving App (Tnfrsf21) [32], underscoring the broad biological scope of the amyloid‐associated proteome captured by Orth‐*i*EA.

To elucidate the relational context of the amyloid interactome, we integrated previously reported and newly identified proteins to construct an expanded PPI network using the STRING database. GO enrichment analysis of the TMGBF_4_‐enriched proteome identified involvement in key AD‐related neuronal processes, such as cell projection morphogenesis, translation, and synaptic transmission (Figure 5D), highlighting Orth‐*i*EA's capture of functionally relevant components. Centering the network on core amyloid hubs such as Tau and App, our analysis revealed that these previously overlooked proteins form extensive interaction clusters with established factors, including Sod1, Fus, Hmgb1, and Pin1 (Figure 5E). This not only validates the amyloid relevance of our discoveries but also demonstrates the unique power of Orth‐*i*EA to bridge known and previously uncharacterized pathological networks, thereby expanding the landscape of the amyloid interactome.

Further functional dissection of these interaction networks revealed distinct biological signatures. Proteins interacting with both Tau and App were enriched in pathways related to axonogenesis and microtubule organization, with predominant localization in neurons and axons, consistent with the roles of Tau and App in axonal transport and synaptic stability (Figure S5A). Tau‐specific interactors were significantly associated with mitochondrial functions (such as ATP synthesis and electron transport), mitochondrial membrane localization, and microtubule binding activity, providing insight into how Tau aggregation might disrupt mitochondrial energy production and induce oxidative stress (Figure S5B). These links further implicate Tau in cellular energy deficits. In contrast, App‐specific interactors were enriched in memory, vesicle fusion, and apoptotic regulation, with localization in synaptic and extracellular compartments, aligning with the known impact of App‐derived species on synaptic toxicity and neurotransmission (Figure S5C).

Collectively, these results indicate that the Orth‐*i*EA strategy enables high‐fidelity isolation and characterization of amyloid aggregates from complex tissue environments. It substantially expands the known amyloid interactome by identifying novel, functionally relevant protein networks implicated in AD pathogenesis. This methodological advance provides a powerful tool for dissecting the molecular complexity of amyloid pathology and may facilitate future discovery of therapeutic targets in neurodegenerative diseases.

## 3 Conclusion

We have developed a novel orthogonal ionic‐liquid extraction system, Orth‐*i*EA, that significantly enhances the depth and specificity of amyloid proteomics. Through systematic screening of IL pairs, TMGBF_4_ was identified as a uniquely potent reagent capable of penetrating extensive hydrogen‐bond networks within cross‑β architectures, thereby eliminating the inefficiencies of conventional detergent‐ or chaotrope‐based extraction. In combination with C12ImCl, which preferentially solubilizes non‐amyloid proteins, Orth‐*i*EA performs a coordinated two‐step enrichment that reduces proteome complexity while preserving low‐abundance amyloid species. This complementary chemistry affords high selectivity and reproducibility, enabling quantitative and depth‐resolved amyloid proteomics. When applied to hippocampal tissue from 3xTg‐AD mice, Orth‐*i*EA uncovered diverse amyloid‐associated proteins, linking amyloid accumulation to mitochondrial dysfunction. Overall, this dual‐IL extraction method advances amyloid proteomics with superior selectivity, sensitivity, and LC‐MS compatibility, enabling analysis of insoluble amyloids from limited samples and supporting biomarker discovery, mechanistic studies, and therapeutic targeting in neurodegenerative diseases.

## 4 Experimental Section

### 4.1 Systematic Screening of ILs for Amyloid Aggregate Extraction

First, the solubility of amyloid Tau‐K18 in various IL cations was examined. Equal aliquots of amyloid Tau‐K18 were solubilized in 100 µL of 1 M ILs solutions, including P1,4Cl, PP1,4Cl, TMGCl, N4,4,4,1Cl, BpyCl, and C4ImCl in PBS (Table S1). The samples were mixed using a vortex mixer. Afterward, the samples were centrifuged at 16,000 ×g for 20 min, and the supernatants were quantified by the BCA assay at 562 nm with BSA as the standard protein. Second, the solubility of amyloid Tau‐K18 in different anions of ILs was evaluated. Equal aliquots of amyloid Tau‐K18 were solubilized in 100 µL of 1 M ILs, including TMGCl, TMGBF_4_, TMGHSO_4_, TMGNO_3_, TMGLac, TMGTFA, TMGOTf, and TMGAc in PBS (Table S1). Samples were processed as described above. Finally, the optimal IL concentration for solubilizing amyloid Tau‐K18 was determined. Equal aliquots of amyloid Tau‐K18 were solubilized in 100 µL of ILs containing 6 m, 8 m, 10 m TMGBF_4_ and 6 m, 8 m, 10 m TMGCl in PBS. All experiments were performed in triplicate.

### 4.2 Solubility Comparison of ILs and Conventional Solvents for Protein Aggregates and Total Cellular Proteins

To evaluate the solubilization efficiency of various solvents on total cellular proteins and protein aggregates, a panel of solvents was prepared in PBS, including ILs (C12ImCl, 10% w/v; TMGBF_4_, 10 m), chaotropes (urea, 8 M; GdnHCl, 6 m), ionic detergents (SDS, 4% w/v; SDC, Sarkosyl, and CHAPS, 1%w/v each) and nonionic detergents (Triton X‐100, NP‐40, 1% v/v each). For total cellular proteins, approximately 10^6^ HEK293 cells were lysed in the above solvents (with 1% v/v protease inhibitor cocktail) and combined with sonication for 2 min (80 W, 5 s on /5 s off pulse). The cell lysate was centrifuged at 16 000 ×g for 20 min, and the protein concentration of supernatants was measured by BCA assay at 562 nm with BSA as the standard protein. For protein aggregates (Amyloid Tau‐K18, DHFR aggregates, and TTR aggregates), equal aliquots of aggregates were resuspended in the indicated solvents. After centrifugation at 20 000 ×g for 20 min, the protein concentration in the supernatants was determined by BCA assay using BSA as the standard. All experiments were performed in triplicate.

### 4.3 Tau‐K18 Cross‐Linking

Equal aliquots of amyloid Tau‐K18 were solubilized in 1%(v/v) NP‐40, 10% (w/v) C12ImCl or 10 m TMGBF_4_. After vortexing and centrifugation at 20 000 ×g for 20 min, supernatants were collected. Native Tau‐K18 in PBS served as the control group. All samples (four groups) were cross‐linked with BS^3^, TDS, or BS(PEG)_2_ at a final concentration of 2 mm. TMGBF_4_ samples were adjusted to pH 7.0 with HEPES before cross‐linking.

### 4.4 Molecular Dynamics Simulations

The initial structure of human Tau‐K18 protein was constructed using the online SWISS‐MODEL platform, with the Tau‐CTE‐MIA11 (PDB ID: 8Q9A) as the template [33, 34]. Tau‐K18 protein appeared as a homotrimer formed by the accumulation of β‐sheets. The solvent molecules (NP‐40, C12ImCl, and TMGBF_4_) were water molecules randomly assembled around the Tau‐K18 protein based on the concentration provided in the experiment using the PACKMOL program to build the systems with the Tau‐K18 protein in different IL environments [35]. Tau‐K18 protein, solvent molecules, and water molecules were assembled into a 150 × 100 × 50 Å^3^ rectangular prism. Detailed parameters and protocols for the MD simulations are provided in the Supporting Information.

### 4.5 Performance Evaluation of the Orth‐*i*EA Strategy

Amyloid Tau‐K18 was labeled with AF594 dye with a final concentration of 0.75 mg/mL at 37°C, 850 rpm for 4 h. After labeling, aggregates were washed with PBS for 5 times to remove the unreacted fluorescent dye. Total cellular proteins and AF594‐labeled amyloid Tau‐K18 were mixed in 10% (w/v) C12ImCl, followed by centrifugation at 16 000 ×g for 5 min. The supernatant was collected, and the precipitate was redissolved in 10% (w/v) C12ImCl. Repeat the procedure 4 times. Each supernatant was collected, and the last precipitate was dissolved in 10 M TMGBF_4_. Protein concentration of the six supernatant samples was measured using the BCA assay at 562 nm with BSA as the standard protein. Fluorescence intensity of the samples was measured on nitrocellulose membranes and imaged using the VISQUE InVivo Smart‐LF bio‐imaging system.

### 4.6 Amyloid Proteomic Profiling in Tissue Using the Orth‐*i*EA Strategy

Three 12‐month‐old female 3xTg‐AD mice were purchased from Shulaibao (Wuhan) Biotechnology Co., Ltd. Hippocampal tissues were homogenized in NP‐40 buffer (1% (v/v) in PBS containing 1% protease and phosphatase inhibitors) using a freezing grinder with 2 mm zirconia grinding beads (4.5 m/s, 40 s on /60 s off, 10 cycles). The homogenates were sonicated on ice (3 min, 80 W, 5 s on /5 s off pulse) and centrifuged at 30 000 ×g for 30 min. The resulting pellet was then sonicated in C12ImCl buffer (10% (w/v) in PBS with 1% protease and phosphatase inhibitors) under the same conditions, followed by centrifugation at 30 000 ×g for 30 min. The supernatant was collected as the “C12ImCl fraction”. The remaining pellet was further sonicated in TMGBF_4_ buffer (10 m in PBS with 1% protease and phosphatase inhibitors), and the resulting supernatant was collected as the “TMGBF_4_ fraction”. Proteins (150 µg per fraction) were denatured with 100 mm DTT (50 mM NH_4_HCO_3_, 56°C, 1 h), loaded onto 10 kDa filters, and alkylated with 20 mM IAA (30 min, dark). Filters were repeatedly washed four times with 50 mM NH_4_HCO_3_, followed by trypsin digestion (6 µg, 37°C, 16 h). Peptides were recovered by centrifugation and eluted with 50 µL water.

### 4.7 Ethical Statement

All animal procedures were conducted in accordance with the “Guiding Principles in the Care and Use of Animals” (China) and were approved by Institutional Animal Care and Use Committee (IACUC) of Wuhan Youdu Biotechnology Co. Ltd., China (Approval Number: 20231128); Shouzheng Hongyao (Wuhan) Biotechnology Co. Ltd., China (Approval Number: 2024101101); Nanjing Jinzhihe Biotechnology Co., Ltd., China (Numbers: IACUC26‐0075 and IACUC26‐0081).

### 4.8 Statistical Analysis

Protein solubility was normalized against the maximum value obtained among the tested solvents and is presented as relative solubility (mean ± SD; n = 3–4). Statistical analyses were performed using GraphPad Prism 8.0.2 software and Origin 2025b software. The overlapping proteins between the TMGBF_4_ and C12ImCl fractions in three replicates were mapped onto a volcano plot by Origin 2025b, and *P*‐values were calculated by two‐sided Student's *t*‐test; ^*^ *p* < 0.05 was considered statistically significant.

## Conflicts of Interest

The authors declare no conflict of interest.

## Supporting information




**Supporting File 1**: advs75405‐sup‐0001‐SuppMat.docx.

**Supporting File 2**: advs75405‐sup‐0002‐DatasetS01.xlsx.

**Supporting File 3**: advs75405‐sup‐0003‐DatasetS02.xlsx.

**Supporting File 4**: advs75405‐sup‐0004‐DatasetS03.xlsx.

**Supporting File 5**: advs75405‐sup‐0005‐DatasetS04.xlsx.

## Data Availability

The data that support the findings of this study are openly available in iProx at https://www.iprox.org, reference number 13836000.
