# Supplementary material for: Orthogonal Ionic Liquid‐Based Extraction Strategy Enables Amyloid‐Specific Profiling of Aggregate Proteome
Source: Adv Sci (Weinh). 2026 Apr 20;13(40):e23685. doi: 10.1002/advs.202523685 (PMC13335754; doi:10.1002/advs.202523685)
Supplement: Supplementary file 1 — Supporting File 1: advs75405‐sup‐0001‐SuppMat.docx. [file ADVS-13-e23685-s004.docx]

Supporting Information

Orthogonal Ionic Liquid-based Extraction Strategy Enables Amyloid-Specific Profiling of Aggregate Proteome

*Shiying Zheng, Ye Liu, Bowen Zhong, Huiying Chu, Zhou Gong, Baofeng Zhao, Mengchun Cheng, Zhen Liang, Yukui Zhang, Qun Zhao*, Lihua Zhang**

1. **Materials and methods**

**1.1. Materials and reagents:** Guanidine hydrochloride (GdnHCl), urea, sodium dodecyl sulfate (SDS), sodium deoxycholate (SDC), N-lauroyl-sarcosine sodium (Sarkosyl), TritonX-100, phosphotungstic acid hydrate, dithiothreitol (DTT), iodoacetamide (IAA), thioflavine-S (ThS), thioflavine-T (ThT), bovine serum albumin (BSA), isopropyl β-D-thiogalactoside (IPTG), imidazole, ammonium bicarbonate (NH_4_HCO_3_), sodium acetate (NaOAc), potassium chloride (KCl), sodium chloride (NaCl), ethylenediaminetetraacetic acid disodium salt dihydrate (EDTA), phosphatase and protease inhibitor cocktail were purchased from Sigma (St. Louis, MO, USA). Tris(hydroxymethyl)aminomethane (Tris), sodium phosphate dibasic heptahydrate (Na_2_HPO_4_·7H_2_O), sodium phosphate monobasic monohydrate (NaH_2_PO_4_·H_2_O), and 3-[(3-Chloamidopropyl) dimethyl-ammonium]-1-propanesulfonate (CHAPS) were purchased from Aladdin Scientific (Shanghai, China). Formic acid (FA, 99.9%) was purchased from Macklin Biochemical Technology (Shanghai, China). Dulbecco's Modified Eagle Medium (DMEM), HEPES buffer solution (1 M), bis(sulfosuccinimidyl)suberate (BS^3^), and OxoidTM Tryptone, OxoidTM Yeast Extract were purchased from Thermo Fisher Scientific (Waltham, MA, USA). Phosphate-buffered saline (PBS) was purchased from Solarbio Science & Technology (Beijing, China). Bicinchoninic acid Assay (BCA) Kit, IGEPAL® CA-630 (Nonidet P-40 Substitute, Reagent grade) and Tween-20 were bought from Beyotime (Shanghai, China). Bis-(PEG)_2_-NHS ester (BS(PEG)_2_) was bought from MedChemExpress (Shanghai, China). Alexa Fluor 594 NHS ester was bought from Duofluor biotechnology (Wuhan, Hubei, China). Heparin was bought from Amsbio Biotechnology (Abingdon, UK). Trypsin gold, Mass Spectrometry Grade was bought from Promega (Madison, WI, USA). Acetonitrile (ACN, HPLC grade) was bought from Merck (Darmstadt, Germany). Deionized water was purified by a Milli-Q system from Millipore (Milford, MA, USA). ReproSil-Pur C18-AQ particles (1.9 μm, 120 Å) was bought from Dr. Maisch Gmbh (Ammerbuch-Entringen, Germany). Fused-silica capillaries (150 µm i.d./375 µm o.d.) were ordered from Sino Sumtech (Handan, Hebei, China). Trehalose disuccinimidyl ester (TDS) was synthesized according to the method previously.^[1]^ The filters with a relative molecular mass cut-off of 3 kDa and 10k Da were purchased from Sartorius AG (Goettingen, Germany). The Oasis HLB extraction cartridges (1cc, 10 mg, 30 μm) were purchased from Waters Corporation (Milford, MA, USA).

**1.2. Antibodies:** Anti-DR6, EIF4G1 antibodies were ordered from Proteintech Group (Wuhan, China). Anti-Tau-5, beta Amyloid 1-40, and ARMET/ARP (MANF) antibodies were ordered from Abcam (Cambridge, UK). Anti NHE-1, TTBK1 antibodies were ordered from Santa Cruz Biotechnology (Santa Cruz, CA, USA). Alexa FluorTM Plus 555 goat anti-Mouse IgG (H+L), Alexa FluorTM 568 donkey anti-rabbit IgG (H+L) were purchased from Thermo Fisher Scientific (Waltham, MA, USA).

**1.3. Ionic liquids (ILs):** P1,4Cl, PP1,4Cl, N4,4,4,1Cl, BpyCl, C4ImCl and 1-dodecyl-3-methylimidazolium chloride (C12ImCl) were purchased from Shanghai Cheng Jie Chemical (Shanghai, China). TMGCl was purchased from Lanzhou Greenchem ILs (Lanzhou, Gansu, China). TMGBF_4_, TMGHSO_4_, TMGNO_3_, TMGLac, TMGTFA, TMGOTf and TMGAc were purchased from Lanzhou Yulu Fine Chemical (Lanzhou, Gansu, China).

1. **Methods**

**2.1. Protein purification:** Tau-K18 was cloned with His-tag at their C-termini for easy purification purpose. The plasmids were transformed into BL21(DE3) E. coli cells. Cells were then grown in Luria Bertani (LB) media and incubated at 37 °C, at 200rpm until the OD600 was in the range of 0.6-0.8. Subsequently, the final concentration of 1 mM IPTG was added and incubated at 37 °C, at 200rpm overnight to induce protein synthesis. Cells were harvested by centrifugation at 3000 ×g for 10 min at 4 ℃ and washed twice with PBS (pH 7.4). After resuspension in buffer A (50.0 mM Tris-HCl, 500 mM NaCl, and 20 mM imidazole, 1mM DTT, pH 8.00), cells were lysed by sonication at 150W for 30min (2s on/off) at 4°C. The supernatant obtained by centrifugation (15,000 ×g, 30 min) was loaded into a Ni-NTA column at 0.5mL/min and then was washed by buffer A at 1 mL·min^-1^. Proteins were eluted by 0-50% buffer B (50.0 mM Tris-HCl, 500 mM NaCl, and 500 mM imidazole,1mM DTT, pH 8.00). And the eluent was identified by SDS-PAGE to determine the elution time of Tau K18 protein. Further purification of proteins was performed on Cytiva HiPrep Desalting column and Cytiva Superdex 75 size-exclusion column in SEC buffer (2.8 mM NaH_2_PO_4_, 7.2 mM Na_2_HPO_4_, 100 mM KCl, 1 mM EDTA, pH 7.4). After the target protein eluent was determined by SDS-PAGE, the eluent was dialyzed with PBS twice. The DHFR, TTR and α-synuclein were gifted by Prof. Yu Liu from Dalian Institute of Chemical Physics, Chinese Academy of Sciences.

**2.2. ThT fluorescence assay:** To monitor Tau-K18 aggregation kinetics, Tau-K18 protein (10-15 μM) was incubated in phosphate buffer (pH 7.40) containing 75 μM ThT, 1 mM DTT and 1.0-1.5 nM heparin at 37 ℃, at 700rpm for 72 h. To monitor α-synuclein aggregation kinetics, α-synuclein (100 μM) was incubated in phosphate buffer (pH 7.40) containing 75 μM ThT, 1 mM DTT at 37 ℃, at 700rpm for 79 h. The ThT fluorescence intensity of protein sample was measured by SpectraMax M2e Multi-Mode Microplate Readers (Molecular Devices) at 444 nm excitation/485 nm emission). The fluorescence intensity was measured every 2 h for the first 12 h of incubation. After incubation for 12 h, the fluorescence intensity was measured every 12h. Each time point was measured three times in each experiment.

**2.3. Preparation of protein aggregates:** For amyloid Tau-K18, Tau-K18 protein (10-15 μM) was incubated in phosphate buffer (pH 7.40) containing 1mM DTT and 1.0-1.5 nM heparin at 37 ℃, at 700rpm for 72 h. For aggregated DHFR, DHFR protein (25.6 μM) was incubated in aggregation buffer (200 mM NaOAc, 100 mM KCl, pH 6.23) at 65 ℃ for 10 min. For aggregated TTR, TTR protein (5.5 μM) was incubated in aggregation buffer (200 mM NaOAc, 100 mM KCl, pH 4.40) at 37 ℃ for 72 h.

**2.4. TEM characterization of protein aggregates:** Tau-K18 aggregates with aggregation time of 12 h, 24 h, 48 h, 72 h were respectively sonicated in water for 1 min (60 W, 2 s on /2 s off pulse) to prepare uniformly dispersed suspensions. Subsequently, samples were pretreated in the negative staining mode, 10 μL of the suspensions and 10 μL of 0.5% (m/v) phosphotungstic acid in water were added to the carbon grid in turn. The images were collected with a JEM-2100 Electron Microscope operated at 200 kV. For DHFR aggregates and TTR aggregates, 10 μL of the suspensions and 10 μL of 0.5% (m/v) phosphotungstic acid in water were added to the carbon grid in turn. The images were collected by JEM-2000EX Electron Microscope at 120 kV.

**2.5. Tau-K18 cross-linking sample preparation**

For the control group, proteins were precipitated with 4 times volume of pre-cooled acetone at −20 °C overnight, washed three times with acetone, and resolubilized in 8 M urea. Samples were further reduced with 10 mM DTT (56 °C, 1 h), alkylated with 20 mM IAA (dark, 30 min), diluted to 1 M urea, and digested with trypsin at an enzyme-to-protein ratio of 1:25 (w/w) at 37 °C for 16 h.

For NP-40, C12ImCl, and TMGBF_4_ groups, samples were reduced with 100 mM DTT, alkylated with 20 mM IAA, and processed on 3 kDa filters. NP-40 samples were washed with 8 M urea and 50 mM NH_4_HCO_3_, while C12ImCl and TMGBF_4_ samples were washed with 50mM NH_4_HCO_3_. Proteins were digested with trypsin at an enzyme-to-protein ratio of 1:25 (w/w) at 37 °C for 16 h. Peptides were collected by centrifugation, and 50 μL of water was added to eluate the remaining peptides on the filters.

**2.6. Evaluation of the solubilization effects of ILs on α-synuclein aggregates:** For aggregation, α-synuclein (200 μM) was incubated in PBS solution containing 1 mM DTT and 75 μM ThT at 37 ℃, at 700rpm for 79 h. α-synuclein aggregates were collected and washed four times with PBS to remove unbound ThT molecules. The protein was subsequently dissolved in equal volume of 10% (w/v) C12ImCl and 10 M TMGBF_4_ in turn. The protein content of the supernatants was quantified by the BCA method at 562 nm with BSA as the standard protein. The ThT fluorescence intensity of the supernatants was measured by SpectraMax M2e Multi-Mode Microplate Readers (Molecular Devices) at 444 nm excitation and 485 nm emission. The experiment was performed in three replicates.

**2.7. Solubility of Tau-K18 aggregates in different aggregation stages:** Tau-K18 aggregates at different aggregation timepoints (12 h, 24 h, 48 h and 72 h) were prepared by equal volume of native Tau-K18 protein. The aggregates at each time point were dissolved by 1% (v/v) NP-40, 10% (w/v) C12ImCl and 10 M TMGBF_4_ in turn. The three supernatants from different aggregation time were then quantified by the BCA method at 562 nm with BSA as the standard protein. The experiment was performed in three replicates.

**2.8. Molecular dynamics simulations:** Atomistic molecular dynamics simulations of Tau-K18 protein with different IL systems were carried out with AMBER16 software, and the force field for Tau-K18 protein was embraced with Amber ff14SB.^[2]^ The electronic potential parameters of NP-40, C12ImCl and TMGBF_4_ were calculated using the Gaussian 09 program with the B3LYP functional under 6-311G* basis set. The partial charges of three molecules were derived using the RESP charge fitted with the antechamber module in Amber 16.^[3]^ Each simulation complex was immersed in a hexahedral solvent box accompanied by TIP3P model for water,^[4]^ which was neutralized with a number of neutralized ions. Energy minimization was conducted by imposing a strong restraint on simulations system and was followed by minimizing whole system for a few thousand steps. Then, a NVT simulation is performed to heat whole system from 0 to 300 K, which was followed by a 5 ns NPT equilibration run.^[5]^ Subsequently, the production run was performed for 1 μs with a time step of 4 fs, and the coordinates for all models were saved every 2 ps (each simulation system was repeated for five times). During the production run, the SHAKE algorithm was employed to constrain all bonds associated with hydrogen atoms. Electrostatic interaction was treated by Particle Mesh Ewald and the cutoff value of nonbonded interactions is set to 9 Å.^[6]^

The binding free energy between Chain A and Chain B/C of Tau-K18 were calculated by Molecular Mechanics Generalized Born Surface Area (MM-GBSA) method base on the 1000 snapshots extracted from the last 200 ns MD trajectory.^[7, 8]^ The total binding energy (
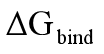
) was computed according to the following equation:


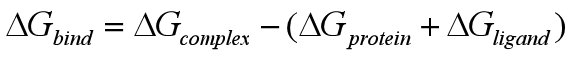
 (1)

where
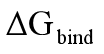
represented the binding free energy between Chain A and Chain B/C, calculated according to the discrepancy between the sum of the free energy of the ligand (
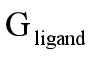
) and the protein (
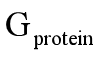
) and the total free energy of the complex (
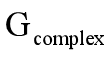
). The binding energy was resented as:


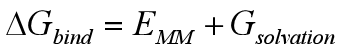
 (2)

where
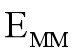
 was the molecular mechanics energy of the molecule expressed as the sum of the internal energy of van der Waals energies and molecule plus electrostatic. The solvation free energy was expressed as nonpolar and polar contributions to the solvation energy:


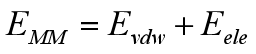
 (3)


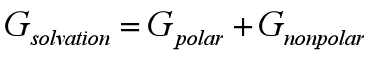
 (4)


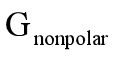
 was calculated from the solvent-accessible surface area (SASA):


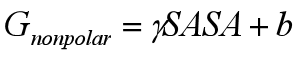
 (5)

where
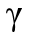
= 0.0072 kcal·mol^-1^·Å^-1^, and
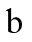
= 0 kcal·mol^-1^.

**2.9 Proteomic profiling of amyloid-positive and amyloid-negative samples using individual IL extraction**

**SH-SY5Y cells**: Equal numbers of SH-SY5Y cells were lysed by sonication (3 min, 80 W, 5 s on / 5 s off) in 200 μL of 10% (w/v) C12ImCl buffer or 200 μL of 10 M TMGBF_4_ buffer in PBS, each containing 1% protease and phosphatase inhibitors. Lysates were centrifuged at 16,000 ×g for 20 min, and the supernatants were collected. Protein concentrations were determined by BCA assay at 562 nm using BSA as the standard. Equal volumes of protein supernatants were denatured with 100 mM DTT (in 50 mM NH_4_HCO_3_, 56 °C, 1 h), loaded onto 10 kDa filters, and alkylated with 20 mM IAA for 30 min in the dark. The filters were washed four times with 50 mM NH_4_HCO_3_, followed by trypsin digestion (protein : trypsin = 25:1, w/w) at 37 ℃ for 16 h. Peptides were recovered by centrifugation and eluted with 50 μL of water. The resulting peptides were lyophilized and analyzed by nano-LC-MS/MS. All samples were processed in triplicate.

**Hippocampal tissues**: Hippocampal tissues were obtained from three 12-month-old female 3xTg-AD mice and three 3-month-old female C57BL/6J mice, purchased from Nanjing Jinzhihe Biotechnology Co., Ltd. Tissues were homogenized in PBS containing 1% protease and phosphatase inhibitors using a freezing grinder with 2 mm zirconia beads (4.5 m/s, 40 s on / 60 s off, 10 cycles). Homogenates were equally divided into two portions and centrifuged at 30,000 ×g for 30 min. The resulting pellets were resuspended in equal volumes of 10% (w/v) C12ImCl buffer or 10 M TMGBF_4_ buffer in PBS, each containing 1% protease and phosphatase inhibitors. The samples were then sonicated on ice for 3 min (80 W, 5 s on / 5 s off) and centrifuged again at 30,000 ×g for 30 min. Supernatants were collected, and protein concentrations were determined BCA assay at 562 nm using BSA as the standard. Equal volumes of protein supernatants were denatured with 100 mM DTT in 50 mM NH_4_HCO_3_ at 56 °C for 1 h, loaded onto 10 kDa filters, and alkylated with 20 mM IAA for 30 min in the dark. The filters were washed four times with 50 mM NH_4_HCO_3_, followed by trypsin digestion (protein : trypsin = 25:1, w/w) at 37 ℃ for 16 h. Peptides were recovered by centrifugation and eluted with 50 μL of water. The resulting peptides were lyophilized and analyzed by nano-LC-MS/MS.

**2.10. LC-MS/MS analysis:**

**Proteome samples:** Peptides were re-dissolved in 0.1% FA in water and subsequently analyzed

using an Easy-nano LC 1200 system coupled with an Orbitrap Exploris 480 mass spectrometer (Thermo Fisher Scientific). For the LC separation, 0.1% FA in water and 80% acetonitrile-0.1%FA in water were used as mobile phase A and mobile phase B. Each sample was analyzed using the following gradient: 0-5 min, 3-8% B; 5-85 min, 8-28% B; 85-102 min, 28-38% B; 102-110 min, 38-100% B; 110-120 min 100% B. For FAIMS separations, the inner and outer electrode temperature were set to 100℃, 4 L·min^-1^ for FAIMS gas flow, and -45/-65 V for compensation voltage. The MS analysis was operated in a data-dependent acquisition (DDA) mode with a resolution of 60000 (m/z = 200) for full MS scans from 350-1500 (m/z) and a resolution of 15000 (m/z = 200) for MS/MS scans. The dynamic exclusion was set to 30 s. The maximum injection time for full MS and MS/MS were 20 ms and 30 ms. The precursors with charge states of 2 to 7 were selected for HCD fragmentation and the HCD collision energy was set as 30%. The mass spectrometry proteomics data have been deposited to the ProteomeXchange Consortium (https://proteomecentral.proteomexchange.org) via the iProX partner repository with the dataset identifier PXD069689.

**Tau-K18 cross-linking samples**: Peptides were re-dissolved in 0.1% FA in water and subsequently analyzed using an Easy-nano LC 1000 system coupled with a Q Exactive mass spectrometer (Thermo Fisher Scientific). For the LC separation, 2% (v/v) acetonitrile-0.1% FA in water and 80% acetonitrile-0.1% FA in water were used as mobile phase A and mobile phase B. Each sample was analyzed at a flow rate of 0.6 μL·min^-1^ using the following gradient: 0-0.1 min, 2-7% B; 0.1-70.1 min, 7-18% B; 70.1-105.1 min, 18-32% B; 105.1-110.1 min, 32-80% B; 110.1-120.1 min 80% B. The MS was operated in the positive-ion mode. Full MS was performed in the scan range of 300-1800 m/z with a resolution of 70,000 and the resolution of MS/MS scans was 17,500. The dynamic exclusion was set to 18 s. For samples cross-linked by TDS, the precursors with charge states of 4 to 10 were selected for HCD fragmentation. The collision energy mode was stepped (N)CE, and the collision energy was set as 25% and 32%. The maximum injection time for MS1 and MS2 were 60 ms and 105 ms. For samples cross-linked by BS^3^ and BS(PEG)_2_, the precursors with charge states of 3 to 10 were selected for HCD fragmentation. The collision energy mode was (N)CE, and the collision energy was set as 28%. The maximum injection time for MS1 and MS2 were both 60 ms.

**2.11. Database searching and data analysis:**

**Proteomic data:** Raw files were processed with pFind 3.2.0 software against the Mouse Uniprot FASTA database (2023-12-15).^[9]^ Trypsin was chosen for the enzyme, and up to 3 missed cleavage sites were permitted. The Carbamidomethyl (C) was set as for the fixed modification. The oxidation on methionine and acetylation of the protein N-terminus were set as the variable modifications. The precursor and fragment tolerance were set to 20 ppm, and the false discovery rate (FDR) for PSM and protein is set at 0.01. Protein intensity values were extracted using pQuant/pGlycoQuant_v202401 software.^[10]^

**Tau-K18 cross-linking data:** Raw files of samples crosslinked by BS^3^ and BS(PEG)_2_ were processed with pLink 2.3.11 and the raw files of samples crosslinked TDS were processed with pLink 3.0.11 against Tau-K18 FASTA (The 243-372 residues of Tau-K18, Uniprot ID: P10636-8).^[11]^ Trypsin was chosen for the enzyme, and up to 3 missed cleavage sites were permitted. The carbamidomethyl (C) was set as for the fixed modification. The oxidation on methionine and acetylation of the protein N-terminus were set as the variable modifications. In addition, the dead-end modification of each cross-linker was set as variable modification (BS^3^, +188.0685 on K; BS(PEG)_2_, +156.0786 on K; TDS, +522.1687 on K). The precursor and fragment tolerance were set to 20 ppm, and the FDR for PSM and protein is set at 0.01. The peptide mass was range from 500−6000 and the peptide length was range from 5-60. K and protein N-term were set as the cross-linking sites, and the mass shift information was as followed: 521.186 for TDS cross-linked mass shift and 522.170 for TDS mono-linked mass shift; 138.068 for BS^3^ cross-linked mass shift and 156.079 for BS^3^ mono-linked mass shift; 170.058 for BS(PEG)_2_ cross-linked mass shift and 188.068 for BS(PEG)_2_ mono-linked mass shift. Merge the cross-linked information from the three cross-linkers.

**Data analysis:** Venn diagrams, volcano plots, heatmaps and principal component analysis (PCA) were performed using Origin2025b software. Bar charts, scatter plots and box plots were performed using the Graghpad 8.0.2 software. Gradient bar chart was generated online using Hiplot (https://hiplot.com.cn/). Gene Ontology (GO) analysis was performed using DAVID (https://david.ncifcrf.gov/) and Metascape.^[12]^ The protein-protein interactions were queried from the STRING database (version 12.0, https://string-db.org/) and mapped by Cytoscape 3.10.3. Protein abundance information was from Protein Abundance Database (Mouse-Brain (Integrated), https://pax-db.org/). The protein abundance scatter plot was generated online using BioLadder.^[13]^ Isoelectric point (pI) and molecular weight (Mw) were computed by the Compute pI/Mw (https://web.expasy.org/compute_pi/). Phase separation score (PS score) was from PhaSePred (Mouse, http://predict.phasep.pro/).^[14]^ Aggregation propensity was from Aggrescan3D Database (https://biocomp.chem.uw.edu.pl/A3D2/MODB).^[15]^ For intrinsically disordered region (IDR) proportion, the corresponding PDB files were downloaded from the AlphaFold database (https://alphafold.ebi.ac.uk/). The secondary structure for each structure was calculated using CPPTRAJ module in AMBER 20 package^[16]^ with Dictionary of Secondary Structure of Proteins algorithm (DSSP).^[17]^ Then, the regions identified as “None” in the secondary structure calculations were designated as IDR regions, and corresponding statistical analyses were performed. Co-aggregation coefficients were calculated by AmyloComp (https://bioinfo.crbm.cnrs.fr/index.php?route=tools&tool=30).^[18]^ Mouse tissue images were obtained from the open-access repository BioGDP.^[19]^ Pearson’s correlation coefficients were calculated using ImageJ.

**2.12. Data validation:** Two of 12-month-old female 3xTg-AD mice and two of 12-month-old female C57BL/6J mice were purchased from Shulaibao (Wuhan) Biotechnology Co., Ltd. All the experiments were performed according to the guidelines of the Chinese Society of Laboratory Animal Sciences to ensure animal welfare. Mouse brain tissue was acquired after the mice were sacrificed by anesthesia. 15 μm thick coronal tissue sections were performed from frozen brain using a cryostat (Leica Biosystems Inc., BuffaloGrove, IL) at -20 ℃ and thaw-mounted onto glass slides. The tissue sections were sliced continuously from one tissue to maintain consistent experimental conditions. The tissue sections were stored at -20 ℃ for subsequent experiments.

Tissue sections were first baked at 60 °C for 30 min, followed by immersion in PBS for 15 min. The area surrounding the tissue was marked using a hydrophobic barrier pen. After air-drying, sections were stained with 1 mg·mL⁻¹ ThS (prepared in 50% ethanol/PBS) in the dark for 15 min. The sections were then rinsed with 40% ethanol/PBS for 1 min, followed by three washes with PBS for 5 min each. Subsequently, the tissue sections were blocked at 37 ℃ with 5%BSA for 1 h. The primary antibody for proteins was diluted in the optimal ratio, applied to the sections, and incubated at 4 °C overnight. After incubation, sections were washed three times with PBST (PBS containing 0.1% (v/v) Tween-20) for 10 min each. Fluorescent secondary antibodies (1:800 dilution) were then applied and incubated at room temperature for 1 h, followed by three additional washes with PBST (10 min each). Finally, sections were mounted with antifade mounting medium and covered with coverslips. Confocal fluorescence imaging was performed using a two-photon confocal microscope system (Olympus Corporation, Japan) with a 40 × objective, using excitation channels at 488 nm and 543 nm.

1. **Supporting figures and tables**

**
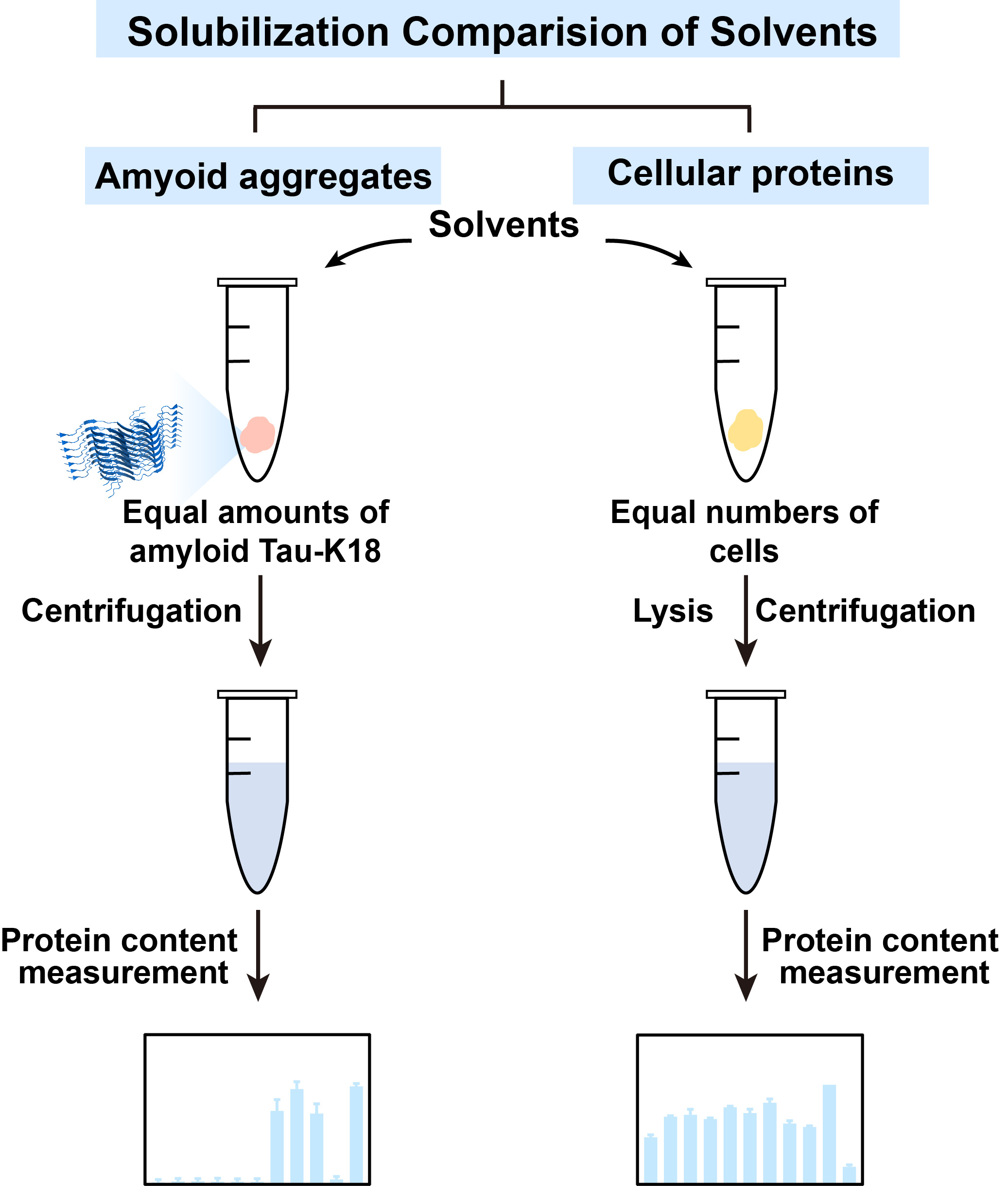
**

**Figure S1.** Schematic workflow for comparing the solubilization of amyloid aggregates and cellular proteins using ILs and conventional solvents.

**
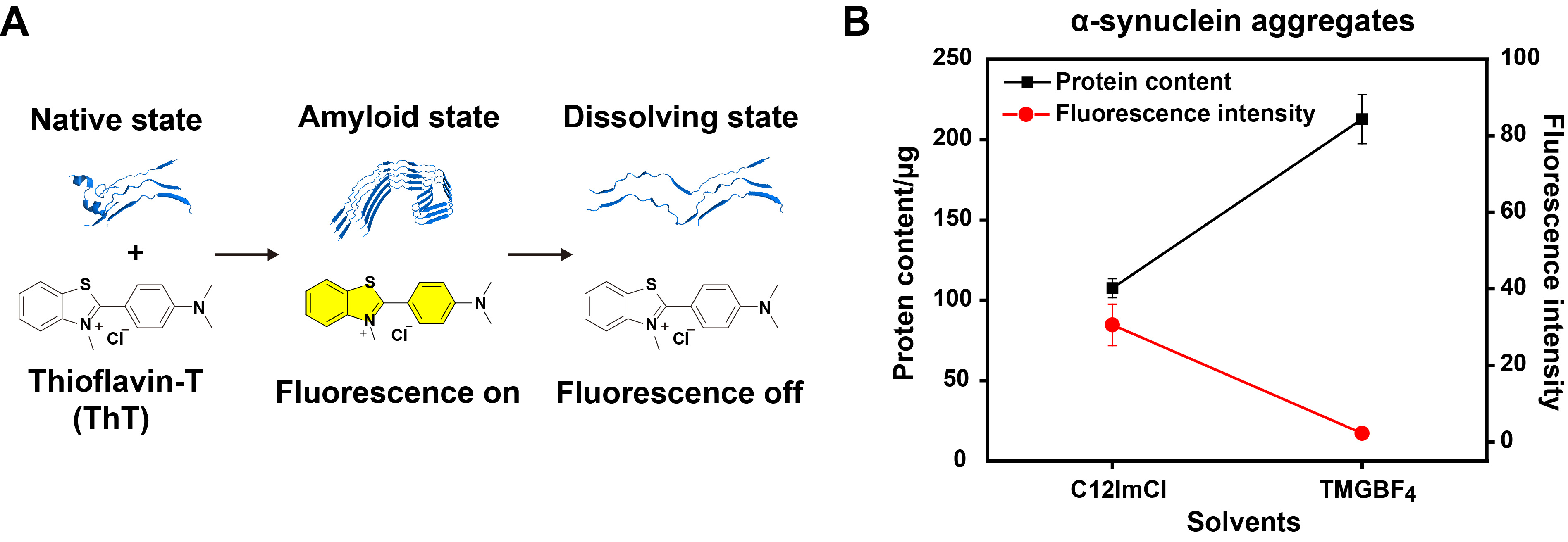
**

**Figure S2.** Evaluation of the solubilization effects of two ionic liquids on α-synuclein aggregates. (A) ThT fluorescence intensity (Ex 444 nm/Em 485 nm) was used to assess β-sheet structures in amyloids. Fluorescence intensity increases in the presence of β-sheet structures and decreases upon their disruption. (B) Investigation on the solubilization effects of two ionic liquids to α-synuclein aggregates. TMGBF_4_ dissolved most of the α-synuclein aggregates with a marked reduction in ThT fluorescence compared to C12ImCl.


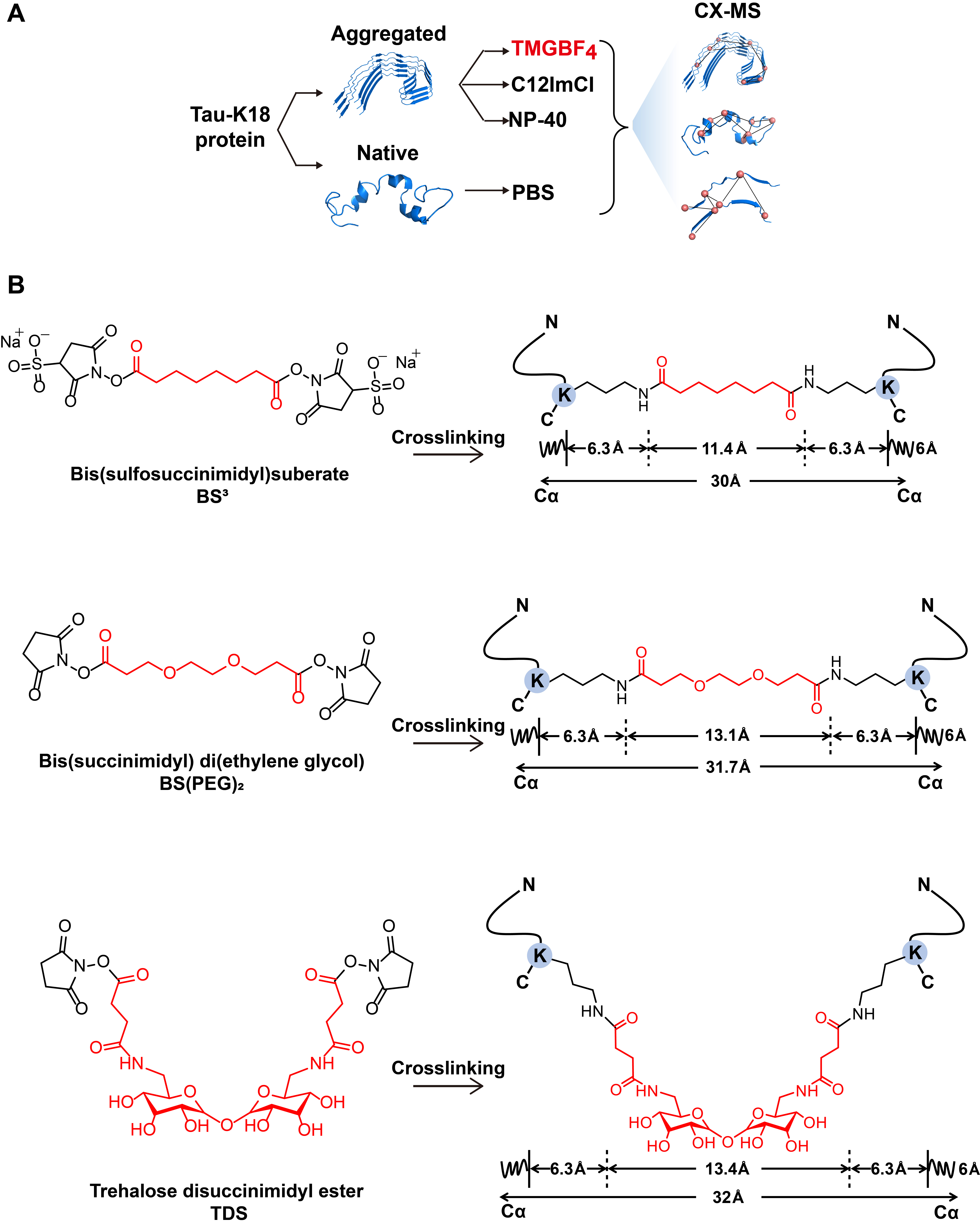
**
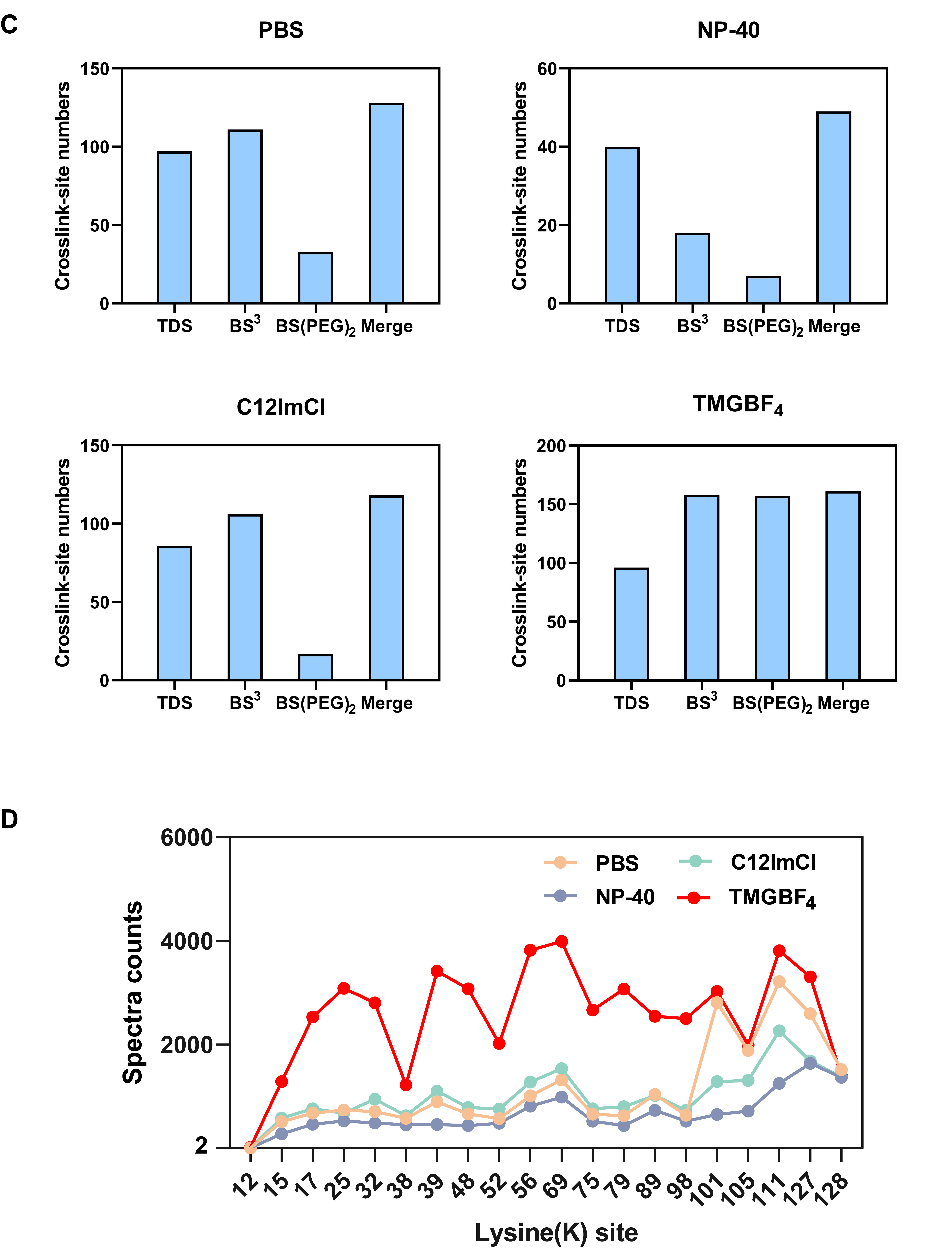
**

**Figure S3.** CX-MS analysis of Tau-K18 in the native state and in aggregated states solubilized by different reagents. (A) Illustration of CX-MS analysis of Tau-K18 in native state, and in aggregated states that solubilized in NP-40, C12ImCl and TMGBF_4_. (B) Three distinct amine-reactive cross-linkers with variable spacer lengths and backbone chemistries were used for the CX-MS experiment. (C) Crosslink-site numbers of native Tau-K18 (in PBS) and amyloid Tau-K18 (solubilized in NP-40, C12ImCl and TMGBF_4_) using different cross-linkers. (D) Comparison on the labeling efficiency of lysine residues (including the spectral numbers of cross-links, loop-links, and mono-links) in PBS, NP-40, C12ImCl, and TMGBF_4_ groups.


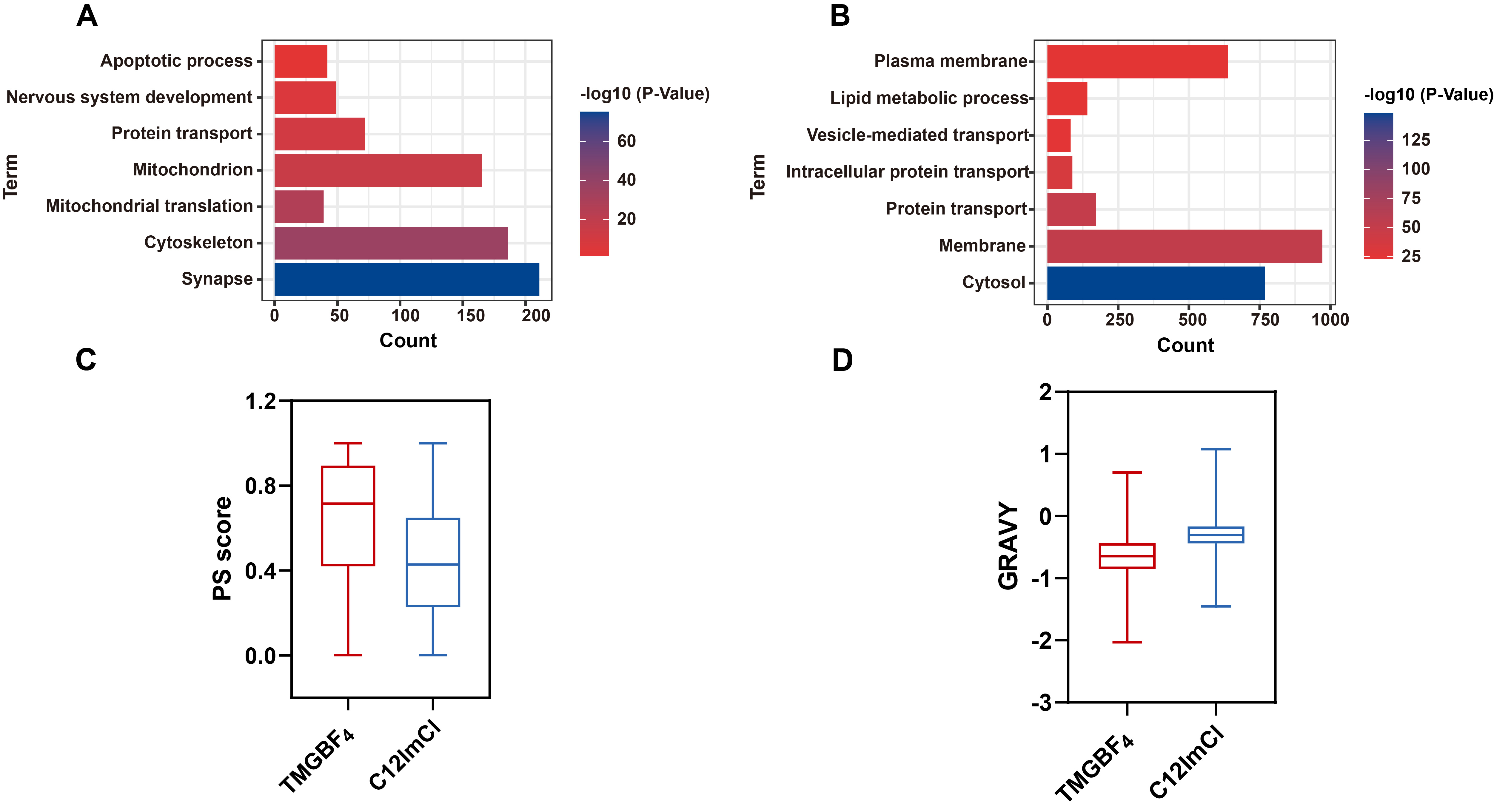


**Figure S4.** Physicochemical and functional characterization of proteins commonly identified in TMGBF_4_ fraction and C12ImCl fraction. (A) GO analysis of TMGBF_4_-enriched proteins. (B) GO analysis of C12ImCl-enriched proteins. (C) PS score distribution of TMGBF_4_-enriched and C12ImCl-enriched proteins. (D) GRAVY value distribution of TMGBF_4_-enriched and C12ImCl-enriched proteins.

**
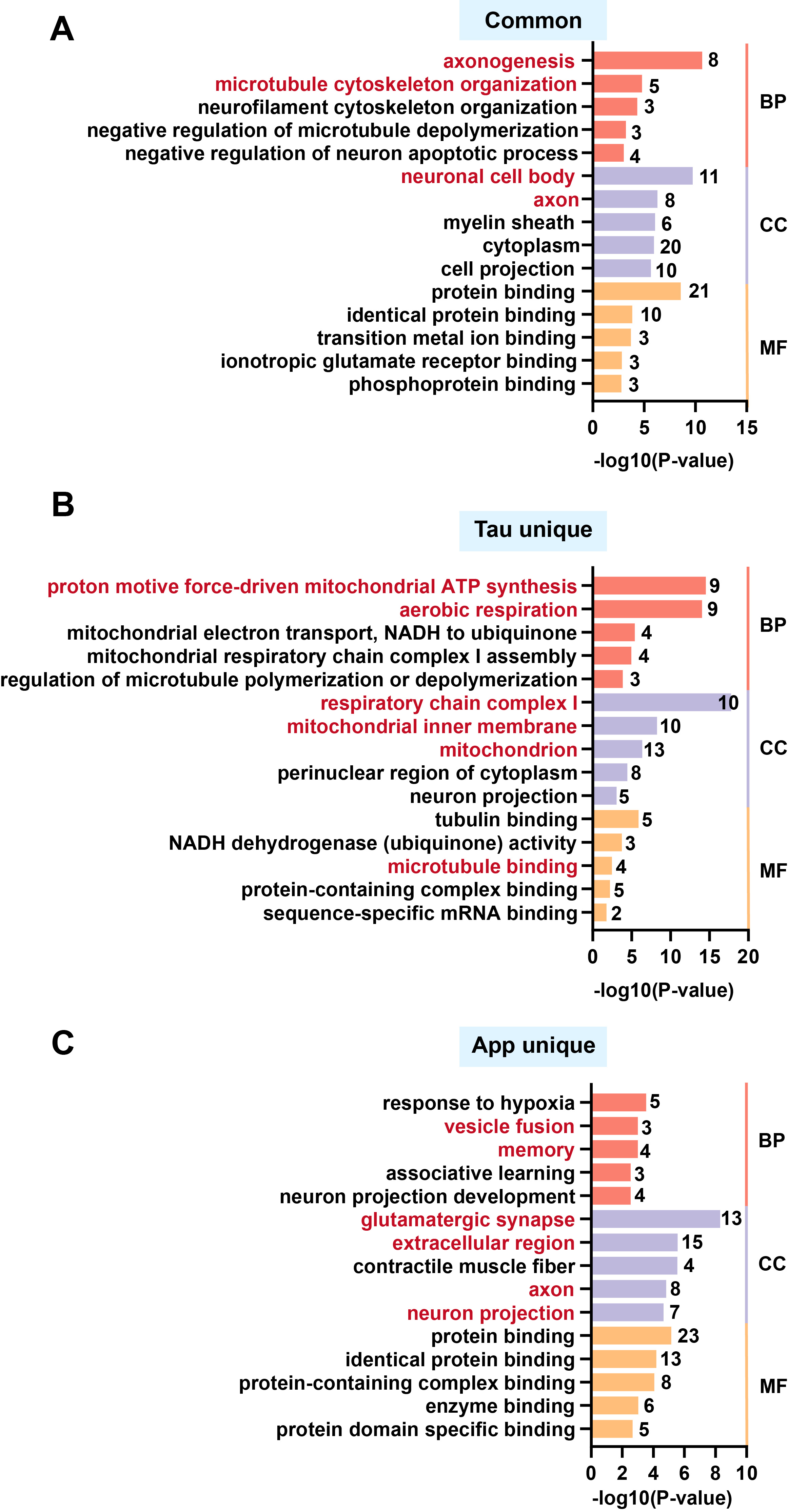
**

**Figure S5.** GO analysis of proteins interacting with Tau or App in TMGBF_4_-enriched proteome. (A) Common interactors of Tau and App; (B) Tau-specific interactors; (C) App-specific interactors.

**Table S1. Screening list of ILs.**

| Name | Formula | Structure |
| --- | --- | --- |
| N-butyl-N-methylpyrrolidinium chloride (P1,4Cl) | C_9_H_20_ClN | 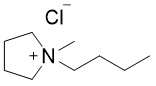 |
| N-butyl-N-methyl-piperidinium chloride (PP1,4Cl) | C_10_H_22_ClN | 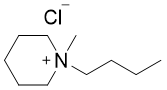 |
| Methyltributylammonium chloride (N4,4,4,1Cl) | C_13_H_30_ClN | 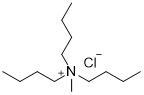 |
| N-butylpyridinium chloride (BpyCl) | C_9_H_14_ClN | 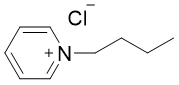 |
| 1-butyl-3-methylimidazolium chloride (C4ImCl) | C_8_H_15_ClN_2_ | 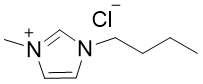 |
| Tetramethylguanidine chloride (TMGCl) | C_5_H_14_ClN_3_ | 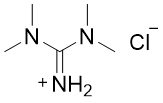 |
| Tetramethylguanidine tetrafluoroborate (TMGBF_4_) | C_5_H_14_BF_4_N_3_ | 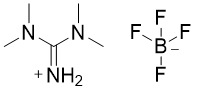 |
| Tetramethylguanidine hydrogensulfate(TMGHSO_4_) | C_5_H_15_N_3_O_4_S | 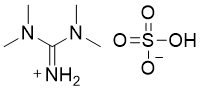 |
| Tetramethylguanidine nitrate (TMGNO_3_) | C_5_H_14_N_4_O_3_ | 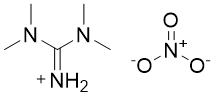 |
| Tetramethylguanidine lactate (TMGLac) | C_8_H_19_N_3_O_3_ | 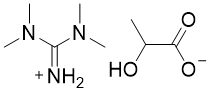 |
| Tetramethylguanidine trifluoroacetate (TMGTFA) | C_7_H_14_F_3_N_3_O_2_ | 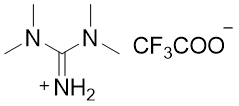 |
| Tetramethylguanidine trifluoromethanesulfonate (TMGOTf) | C_6_H_14_F_3_N_3_O_3_S | 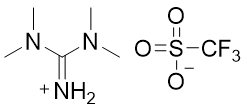 |
| Tetramethylguanidine acetate (TMGAc) | C_7_H_17_N_3_O_2_ | 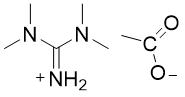 |

**Table S2.** Binding free energy between Chain A and Chain B/C of Tau-K18 homotrimer in different solvents.

| Solvents | Binding free energy  (kcal·mol^-1^) |
| --- | --- |
| Control (in PBS) | -292.33 |
| NP-40 | -283.08 |
| C12ImCl | -285.45 |
| TMGBF_4_ | -274.39 |

**Dataset S01 (separate file).** Aggregation propensity of proteins enriched in TMGBF_4_ extracts from 3xTg-AD and wild-type mouse hippocampus.

**Dataset S02 (separate file).** TMGBF_4_-enriched dataset.

**Dataset S03 (separate file).** GO-Biological Process analysis of the newly identified mitochondrial proteins in the TMGBF_4_ enriched dataset.

**Dataset S04 (separate file).** Protein-protein interaction network analysis of TMGBF_4_-enriched proteins by STRING database.

**SI References**

[1] J. Chen, Q. Zhao, H. Gao, et al., A Glycosidic-Bond-Based Mass-Spectrometry-Cleavable Cross-linker Enables In Vivo Cross-linking for Protein Complex Analysis. *Angew Chem Int Ed Engl* **2023**, *62*, e202212860.

[2] J. A. Maier, C. Martinez, K. Kasavajhala, et al., ff14SB: Improving the Accuracy of Protein Side Chain and Backbone Parameters from ff99SB. *Journal of Chemical Theory and Computation* **2015**, *11*, 3696-3713.

[3] D. Case, Betz, R., Cerutti, D.S., Cheatham, T., Darden, T., Duke, R., Giese, T.J., Gohlke, H., Goetz, A., Homeyer, N., et al. , Amber 2016 (San Francisco: University of California). **2016**.

[4] W. L. Jorgensen, J. Chandrasekhar, J. D. Madura, R. W. Impey, M. L. Klein, Comparison of simple potential functions for simulating liquid water. *J. Chem. Phys.* **1983**, *79*, 926-935.

[5] H. J. C. Berendsen, J. P. M. Postma, W. F. van Gunsteren, A. DiNola, J. R. Haak, Molecular dynamics with coupling to an external bath. *J. Chem. Phys.* **1984**, *81*, 3684-3690.

[6] T. A. Darden, D. M. York, L. G. J. J. o. C. P. Pedersen, Particle mesh Ewald: An N⋅log(N) method for Ewald sums in large systems. *J. Chem. Phys.* **1993**, *98*, 10089-10092.

[7] P. A. Kollman, I. Massova, C. Reyes, et al., Calculating Structures and Free Energies of Complex Molecules:  Combining Molecular Mechanics and Continuum Models. *Accounts of Chemical Research* **2000**, *33*, 889-897.

[8] L. Xu, H. Sun, Y. Li, J. Wang, T. Hou, Assessing the Performance of MM/PBSA and MM/GBSA Methods. 3. The Impact of Force Fields and Ligand Charge Models. *J. Phys. Chem. B.* **2013**, *117*, 8408-8421.

[9] H. Chi, C. Liu, H. Yang, et al., Comprehensive identification of peptides in tandem mass spectra using an efficient open search engine. *Nature Biotechnology* **2018**, *36*, 1059-1061.

[10] S. Kong, P. Gong, W.-F. Zeng, et al., pGlycoQuant with a deep residual network for quantitative glycoproteomics at intact glycopeptide level. *Nature Communications* **2022**, *13*, 7539.

[11] Z.-L. Chen, J.-M. Meng, Y. Cao, et al., A high-speed search engine pLink 2 with systematic evaluation for proteome-scale identification of cross-linked peptides. *Nature Communications* **2019**, *10*, 3404.

[12] Y. Zhou, B. Zhou, L. Pache, et al., Metascape provides a biologist-oriented resource for the analysis of systems-level datasets. *Nature Communications* **2019**, *10*, 1523.

[13] Y. Zhang, C. Yang, J. Wang, et al., BioLadder: A bioinformatic platform primarily focused on proteomic data analysis. *iMeta* **2024**, *3*, e215.

[14] Z. Chen, C. Hou, L. Wang, et al., Screening membraneless organelle participants with machine-learning models that integrate multimodal features. *Proceedings of the National Academy of Sciences* **2022**, *119*, e2115369119.

[15] A. E. Badaczewska-Dawid, A. Kuriata, C. Pintado-Grima, et al., A3D Model Organism Database (A3D-MODB): a database for proteome aggregation predictions in model organisms. *Nucleic Acids Research* **2024**, *52*, D360-D367.

[16] D. A. Case, K. Belfon, I. Ben-Shalom, et al., Amber 2020: University of california. **2020**.

[17] W. Kabsch, C. Sander, Dictionary of protein secondary structure: Pattern recognition of hydrogen-bonded and geometrical features. *Biopolymers* **1983**, *22*, 2577-2637.

[18] S. A. Bondarev, M. V. Uspenskaya, J. Leclercq, et al., AmyloComp: A Bioinformatic Tool for Prediction of Amyloid Co-aggregation. *Journal of Molecular Biology* **2024**, *436*.

[19] S. Jiang, H. Li, L. Zhang, et al., Generic Diagramming Platform (GDP): a comprehensive database of high-quality biomedical graphics. *Nucleic Acids Research* **2025**, *53*, D1670-D1676.
